# Bendless is essential for PINK1-Park mediated Mitofusin degradation under mitochondrial stress caused by loss of *LRPPRC*

**DOI:** 10.1371/journal.pgen.1010493

**Published:** 2023-04-25

**Authors:** Rajit Narayanan Cheramangalam, Tarana Anand, Priyanka Pandey, Deepa Balasubramanian, Reshmi Varghese, Neha Singhal, Sonal Nagarkar Jaiswal, Manish Jaiswal

**Affiliations:** 1 Tata Institute of Fundamental Research, Hyderabad, India; 2 CSIR–Centre For Cellular and Molecular Biology, Hyderabad, India; The University of North Carolina at Chapel Hill, UNITED STATES

## Abstract

Cells under mitochondrial stress often co-opt mechanisms to maintain energy homeostasis, mitochondrial quality control and cell survival. A mechanistic understanding of such responses is crucial for further insight into mitochondrial biology and diseases. Through an unbiased genetic screen in *Drosophila*, we identify that mutations in *lrpprc2*, a homolog of the human *LRPPRC* gene that is linked to the French-Canadian Leigh syndrome, result in PINK1-Park activation. While the PINK1-Park pathway is well known to induce mitophagy, we show that PINK1-Park regulates mitochondrial dynamics by inducing the degradation of the mitochondrial fusion protein Mitofusin/Marf in *lrpprc2* mutants. In our genetic screen, we also discover that Bendless, a K63-linked E2 conjugase, is a regulator of Marf, as loss of *bendless* results in increased Marf levels. We show that Bendless is required for PINK1 stability, and subsequently for PINK1-Park mediated Marf degradation under physiological conditions, and in response to mitochondrial stress as seen in *lrpprc2*. Additionally, we show that loss of *bendless* in *lrpprc2* mutant eyes results in photoreceptor degeneration, indicating a neuroprotective role for Bendless-PINK1-Park mediated Marf degradation. Based on our observations, we propose that certain forms of mitochondrial stress activate Bendless-PINK1-Park to limit mitochondrial fusion, which is a cell-protective response.

## Author summary

Mitochondria are dynamic organelles—they fuse and divide, and thereby change their shape and size. Mitochondrial shape and size are in turn dictated by the physiological need of a cell. The difference in shape and size has been shown to have a profound impact on mitochondrial metabolic capabilities and overall cellular physiology. In this work, we studied the regulation of mitochondrial dynamics in *Drosophila lrpprc2* mutant, which serves as a fly model to study Leigh syndrome—a neurometabolic disease caused by mitochondrial dysfunction. *lrpprc2* mutant flies show an increased number of globular mitochondria in contrast with the tubular network in healthy flies. Importantly, a mitochondrial quality control mechanism inhibits the fusion of globular mitochondria and thus they remain segregated from each other. Suppression of the mitochondrial quality control mechanism results in large globular mitochondria entering the mitochondrial network, however, with a detrimental impact on the viability of cells. Overall, this study highlights the existence of protective responses which act by altering mitochondrial dynamics under mitochondrial stress.

## Introduction

Mitochondria are dynamic organelles and their size varies in response to various cellular cues such as developmental [1] and stress signals [2]. This change in mitochondrial size is crucial for cellular adaptations. For example, cellular stress due to amino acid deprivation [3] or oxidative stress leads to mitochondrial hyperfusion [4]. This form of stress-induced mitochondrial hyperfusion (SIMH) is beneficial as it improves ATP production [5] and protects mitochondria from autophagy [3]. Several mitochondrial disease linked mutations have been shown to alter mitochondrial morphology such as mutations in *COX10* [6] and *TFAM* [7] show accumulation of enlarged mitochondria, possibly due to SIMH, however the mechanism of such responses remain elusive. On the other hand, mitochondrial fragmentation occurs as a beneficial process under different cellular signals. For example, increased mitochondrial fission allows for clearance of damaged mitochondria in embryonic stem cells providing them increased resistance to apoptotic signals [8]. Mitochondrial fission enables segregation of damaged mitochondria to enable their removal through mitophagy [9–13], as well as reduced ROS production [14] and promoting cell survival. Hence, while a normal balance of fission-fusion exists physiologically, a change in metabolic needs or other forms of stress can tilt the balance towards either one process and this generally is required to trigger an adaptive cellular response.

Changes in mitochondrial shape and size, i.e., mitochondrial dynamics, requires regulation of GTPases essential for mitochondrial dynamics. While the Dynamin 1-like (DNM1/Drp1) protein mediates fission, Mitofusins (Mfn1 and Mfn2 in mammals, Marf in *Drosophila*) and Optic Atrophy 1 (OPA1) mediate the fusion of mitochondrial outer and inner membranes, respectively. Several post-translational modifications, such as phosphorylation, acetylation and ubiquitination are crucial for the activity of these proteins, and thereby play an important role in determining mitochondrial size [15,16]. Indeed misregulation of mitochondrial dynamics proteins—Mitofusin, Opa1 or Drp1 are all associated with metabolic and neurodegenerative diseases [17].

The E3 ubiquitin ligase Parkin (Park in *Drosophila*, PARK2 in humans) and the kinase PINK1, which are linked to autosomal recessive early-onset Parkinsonism, are known to regulate mitochondrial quality control [18]. Studies in human cancer cell lines have shown that dissipation of the mitochondrial membrane potential (MMP), increased oxidative stress or mitochondrial unfolded protein response (UPR^mt^) results in the stabilization of PINK1 on the outer mitochondrial membrane (OMM). PINK1 stabilization on the OMM leads to Park recruitment, polyubiquitination of OMM proteins and mitophagy [10,19–21]. Several *in vivo* studies have also shown a conserved role for PINK1-Park in mitophagy [22–29]. While PINK1-Park mediated mitophagy has been extensively studied in cells, how the PINK1-Park pathway is activated under physiological conditions *in vivo* remains elusive [30]. Additionally, *in vivo* studies suggest a pro-fission role of PINK1-Park [31–36], perhaps through the turnover of Mitofusin levels as loss of *Pink1* or *Park* shows increased Marf levels [10,37]. As most of these studies utilize *PINK1* and *PARK2* mutants to study defects in mitochondrial dynamics, the mechanism by which they are regulated *in vivo* under various physiological conditions remains unresolved. Additionally, it is unclear as to how the PINK1-Park pathway may activate mitophagy, alter mitochondrial dynamics or selectively target certain OMM proteins in response to various cellular cues.

To study the regulation of Mitofusin/Marf *in vivo*, we undertook an unbiased genetic mosaic screen in *Drosophila*. Through this screen, we discovered that mutations in *lrpprc2* (referred as *ppr* in figures) and *bendless* (*ben*) causes downregulation and upregulation of Marf levels respectively. *Lrpprc2* is a homolog of human *LRPPRC* that is required for mitochondrial mRNA stability and translation and thus mutations in *LRPPRC* affect mitochondrial oxidative phosphorylation [38,39]. Loss of *LRPPRC* results in a neurometabolic disorder—French-Canadian Leigh Syndrome [40,41]. Studies in worms, mouse and human cells have also shown that loss of *LRPPRC* is associated with large mitochondrial size [42,43]. We found that loss of *lrpprc2* results in proteasome-mediated Marf degradation in a PINK1-Park dependent mechanism. Further, we also discovered that mutations in *bendless* (*ben*), which encodes a K63-linked E2 ubiquitin conjugase, is essential for Marf degradation in *lrpprc2* mutants. We additionally demonstrate an essential role for Ben in regulating PINK1 stability, which in turn is required for maintaining steady state Marf levels in healthy cells. Finally, we show that in *lrpprc2* mutants, Ben suppresses excessive mitochondrial fusion and prevents neuronal death under mitochondrial stress.

## Results

### Loss of *lrpprc2* results in reduced Marf levels

To identify novel regulators of mitochondrial dynamics, we performed a blind screen using a collection of *Drosophila* EMS induced X-chromosome lethal mutations [44,45]. This collection was initially generated to identify mutants with neurodegenerative phenotypes and has previously uncovered mutations in *Marf* [46] and several other genes required for mitochondrial function [39,47]. We screened these mutants for misregulation of Marf protein using an HA-tagged Marf genomic construct (*Marf*::*HA*). We used the *FLP*-FRT mediated mitotic recombination strategy to create mutant clones (non-GFP cells) in a heterozygous background (GFP expressing cells) in the developing wing disc epithelium [48] (S1A–S1A’ Fig). This allowed us to compare Marf levels in mutant and wildtype cells within the same tissue.

**Fig 1:**
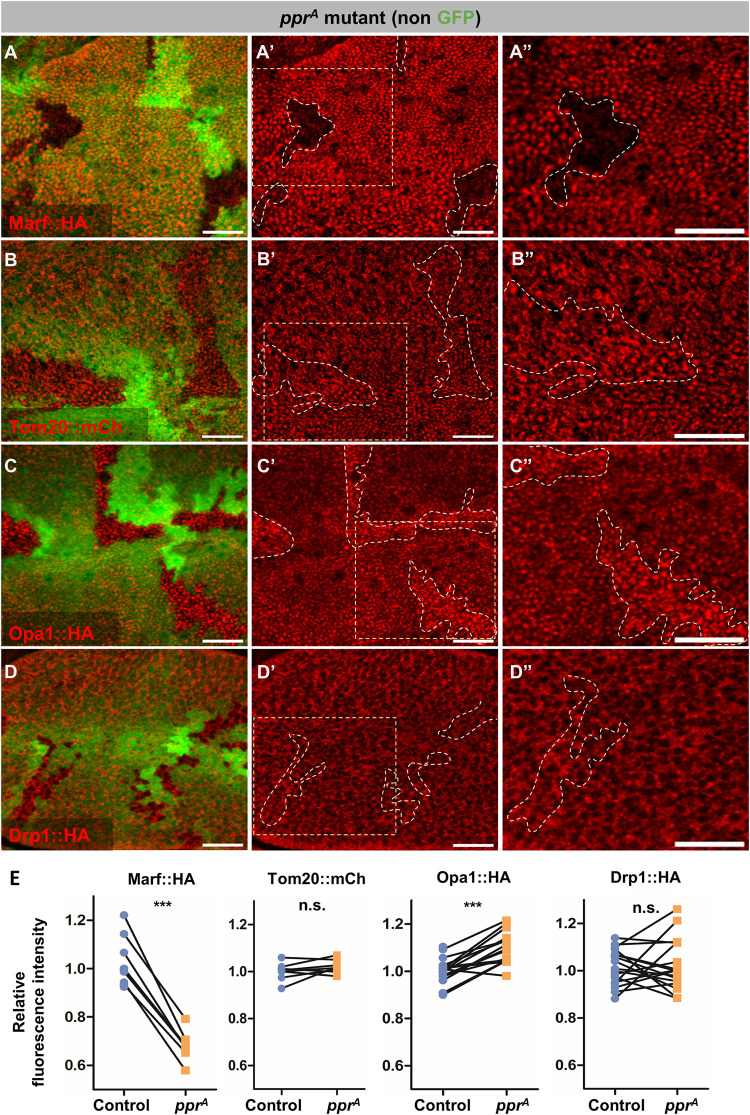
*lrpprc2* mutants show Marf downregulation. *(A-D”) lrpprc2*^*A*^ mutant clones (non green cells, *A-D* and dashed white line, *A’-D’* and *A”-D”)*, wing discs immunostained for Marf::HA (red, *A-A”*), Tom20::mCherry (red, *B-B”*), Opa1::HA (red, *C-C”*) and Drp1::HA (red, *D-D”*) (genomic rescue tags). *A”-D”* are magnified images of insets shown in *A’-D’*. Scale bar represents 20μm. *(E)* Quantification for relative fluorescence intensities of Marf::HA (n = 8), Tom20::mCherry (n = 8), Opa1::HA (n = 16) and Drp1::HA (n = 17) in *lrpprc2*^*A*^ mutant clones. Graphs represent average intensity values normalized to that of control cells. Two-tailed unpaired t-test between control and *lrpprc2*^*A*^ mutant cells. Significance represented by p<0.001***, n.s.—non significant.

From this screen, we found that mutant clones of two *lrpprc2* alleles (*lrpprc2*^*A*^ and *lrpprc2*^*E*^) show reduced Marf:HA levels compared to surrounding wildtype cells (Figs 1A–1A”, 1E and S1B–S1B”). To confirm this observation, we used an independent Marf genomic rescue line, Marf::mCherry, and found reduced Marf::mCherry staining in *lrpprc2*^*A*^ mutant clones (S1C–S1C” Fig). To test if the reduction in Marf::HA or Marf::mCherry is caused by reduced mitochondrial content, we checked the levels of an OMM protein Tom20 using an endogenous tagged line (Tom20::mCherry). We did not observe a downregulation of Tom20::mCherry staining in *lrpprc2*^*A*^ mutant clones (Fig 1B–1B” and 1E). Taken together, these data suggest that downregulation of Marf in *lrpprc2* mutants is not due to reduced mitochondrial content. Additionally, we also checked the levels of other proteins involved in mitochondrial dynamics—Opa1 and Drp1—using genomic tags. While we found the levels of Opa1::HA to be slightly increased in *lrpprc2*^*A*^ mutant clones (Fig 1C–1C” and 1E), Drp1::HA levels remained unaltered (Fig 1D–1 D” and 1E). As mutations in *lrpprc2/LRPPRC* result in mitochondrial defects due to reduced stability of mtRNA [38,39,49], Marf reduction in *lrpprc2* mutants could be an adaptation to segregate defective mitochondria by suppressing their fusion.

Since reduced Marf is expected to suppress mitochondrial fusion, we decided to check mitochondrial morphology in *lrpprc2*^*A*^ mutants. The cells in the wing discs are very compact making it difficult to analyze mitochondrial morphology. Hence, we checked mutant clones in the peripodial membrane, which is a squamous epithelium overlying wing discs. We used anti-Complex V staining to mark mitochondria. Interestingly, we found that mitochondrial size is increased in *lrpprc2*^*A*^ mutant clones (S1D–S1D” and S1E Fig). This is consistent with earlier findings that showed enlarged mitochondrial size in *LRPPRC* knockdown in mouse liver [42], *C*.*elegans* and mammalian cell culture [43]. As many studies have shown that mitochondrial stress can result in increased mitochondrial size [3,5,43,50], we suspect a similar mechanism results in increased mitochondrial size in *lrpprc2* mutant cells, while an independent mitochondrial quality control mechanism may suppress their fusion by reducing Marf levels.

### UPS dependent Marf degradation in *lrpprc2* mutants

**Fig 2:**
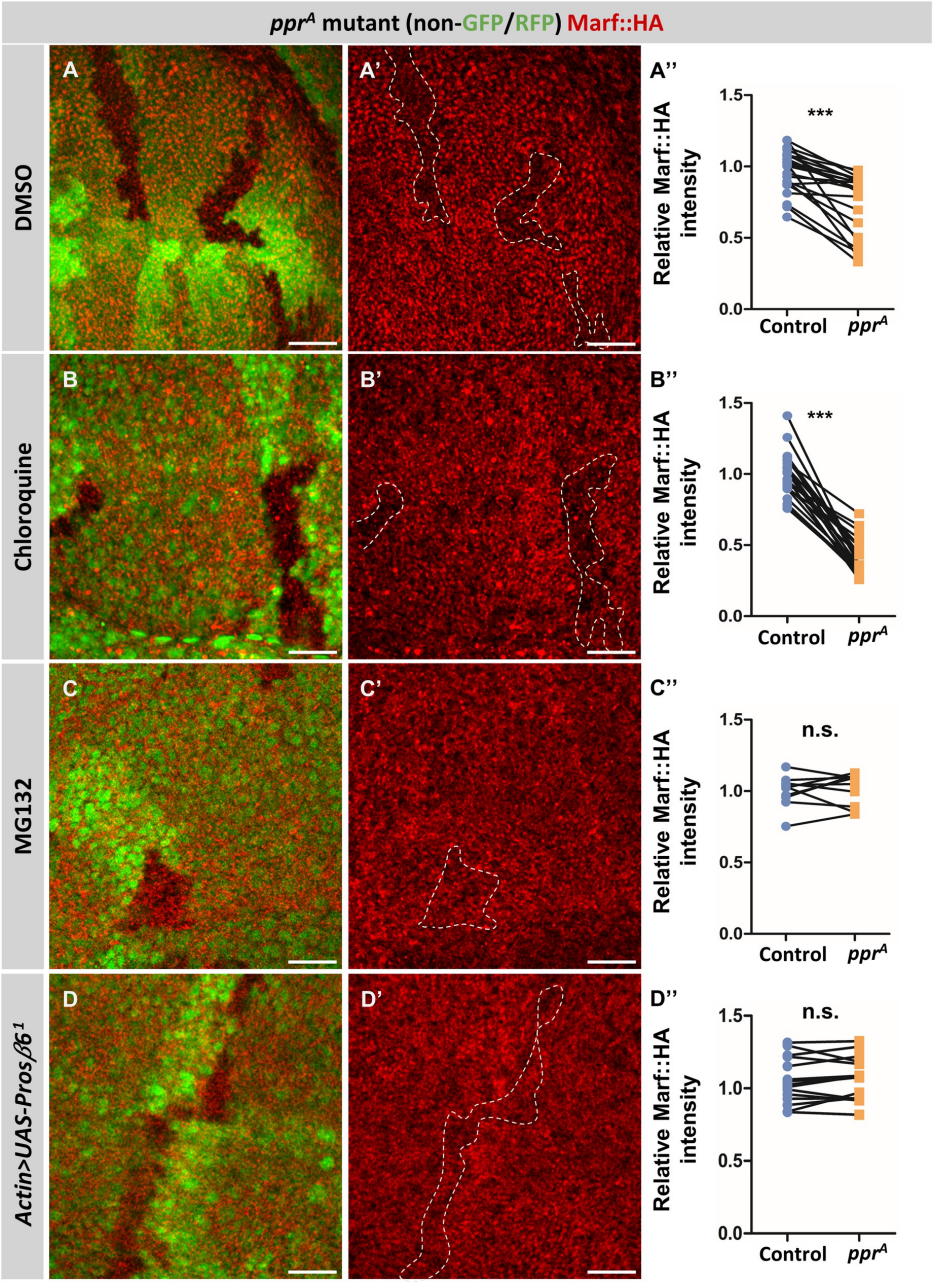
*lrpprc2* mutants show UPS mediated Marf degradation. *(A-C’) lrpprc2*^*A*^ mutant clones (non green cells, *A*, *B* and *C* and dashed white line, *A’*, *B’* and *C’*), wing discs immunostained for Marf::HA (red) after feeding larvae with DMSO*(A-A’)*, chloroquine*(B-B’)* or MG132*(C-C’)*. *(D-D’) lrpprc2*^*A*^ mutant clones (non green cells, *D* and dashed white line, *D’*) on overexpression of *Prosβ6*^*1*^ under *Actin*>*Gal4*, wing discs immunostained for Marf::HA (red). Scale bar represents 20μm. *(A”*, *B”*, *C”* and *D”)* Quantification for relative fluorescence intensities of Marf::HA in *lrpprc2*^*A*^ mutant clones on treatment with, DMSO (*A”*,n = 24), chloroquine (*B”*,n = 23), MG132 (*C”*, n = 10) and on overexpression of *Prosβ6*^*1*^ under *Actin*>*Gal4* (*D”*,n = 15). Graphs represent intensity values normalized to that of control cells. Two tailed unpaired t-test between control and *lrpprc2*^*A*^ mutant cells. Significance represented by n.s. non significant, p<0.001***.

Reduced Marf levels in *lrpprc2* mutant clones could be because of increased protein turnover via selective autophagy or ubiquitin-proteasomal system (UPS). We tested the possibility of autophagic degradation of Marf. We fed the larvae chloroquine, an inhibitor of autophagosome-lysosome fusion [51], and found that Marf::HA levels remain reduced in *lrpprc2*^*A*^ clones (Fig 2B–2B”). To check whether chloroquine treatment itself alters Marf levels, we check endogenous Marf::mCherry levels in wing discs of chloroquine fed larvae and untreated larvae. We observe no effect of chloroquine treatment on Marf::mCherry levels (S2A–S2A” Fig). We found that the levels of p62, a protein degraded primarily via autophagy, was also not altered in *lrpprc2*^*A*^ mutant clones in larval wing discs (S2C–S2C” Fig). Thus we conclude that autophagy is neither enhanced nor likely the cause of Marf reduction in *lrpprc2* mutant clones.

To investigate the role of UPS in Marf downregulation in *lrpprc2* mutants, we fed the larvae with the proteasomal inhibitor MG132 [52,53]. MG132 treatment by itself leads to a subtle increase (Mean intensity normalized to DMSO treated cells—1.203 ± 0.06015) in endogenous Marf::mCherry levels which could be owing to reduced basal turnover of Marf by UPS (S2B–S2B” Fig). In *lrpprc2*^*A*^ mutant clones of DMSO fed larvae, Marf:HA levels were low as compared to the neighboring wildtype cells. However, MG132 fed larvae show no change in Marf::HA levels between wildtype and *lrpprc2*^*A*^ mutant clones (Fig 2A–2A” and 2C–2C”). We further expressed a dominant negative form of *Prosβ6* to inhibit UPS activity [54] and tested its effect on Marf::HA levels in *lrpprc2*^*A*^ mutant clones. Similar to MG132 treatment, we found that Marf::HA levels were restored in *lrpprc2*^*A*^ clones upon *Prosβ6*^*1*^ overexpression (Fig 2D–2D”). These results suggest UPS-mediated degradation of Marf results in Marf reduction in *lrpprc2*^*A*^ clones.

### PINK1 and Park dependent *Marf* regulation in *lrpprc2* mutants

**Fig 3:**
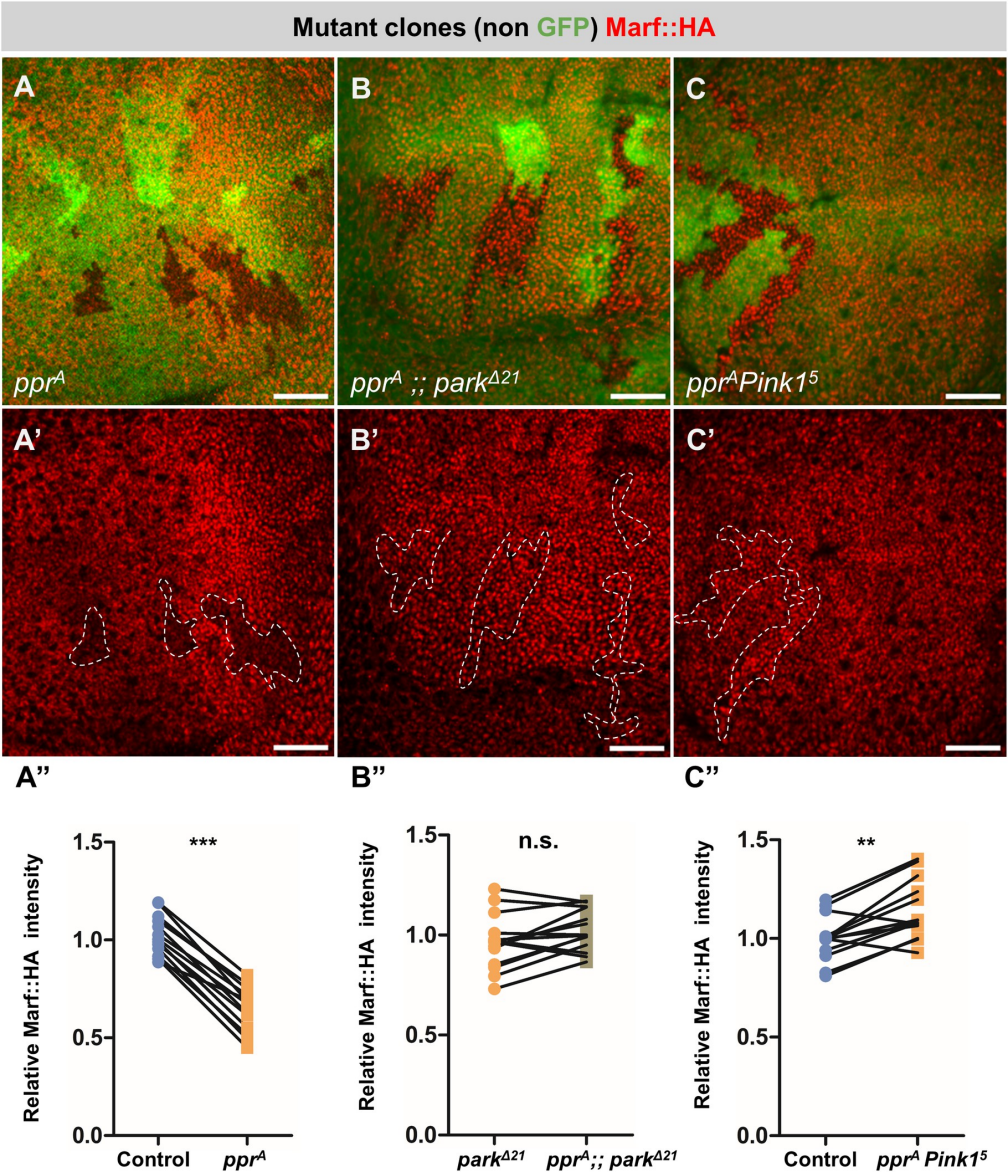
PINK1-Park are required for Marf degradation in *lrpprc2* mutants. *(A-A’) lrpprc2*^*A*^ mutant clones (non green cells, *A* and dashed white line, *A’*), wing discs immunostained for Marf::HA (red). *(B-B’) lrpprc2*^*A*^ mutant clones (non green cells, *B* and dashed white line, *B’*) in *park*^*Δ21*^ background, wing discs immunostained for Marf::HA (red). *(C-C’) lrpprc2*^*A*^ *Pink1*^*5*^ double mutant clones (non green cells, *C* and dashed white line, *C’*), wing discs immunostained for Marf::HA (red). Scale bar represents 20μm. *(A”*, *B” and C”)* Quantification for relative fluorescence intensities of Marf::HA in *lrpprc2*^*A*^ mutant clones (*A”*, n = 13), *lrpprc2*^*A*^ mutant clones in *park*^*Δ21*^ background (*B”*, n = 14) and *lrpprc2*^*A*^ *Pink1*^*5*^ double mutant clones (*C”*, n = 13). Graphs represent average intensity values normalized to that of control/*park*^*Δ21*^ cells. Two-tailed unpaired t-test between control/*park*^*Δ21*^ and mutant cells. Significance represented by n.s.—non significant, p<0.01**, p<0.001***.

Several E3 ubiquitin ligases have been linked to Mitofusin degradation. For example, Mitofusin degradation by HUWE1 occurs under genotoxic stress or under altered fat metabolism conditions [55,56], while Mitofusin degradation by Park occurs on mitochondrial membrane depolarization [9,11]. In *Drosophila* too, HUWE1, MUL1 and Park have been shown to degrade Marf [37,55,57]. On testing these E3 candidates we found a downregulation of Marf::HA levels in *lrpprc2*^*A*^ *HUWE1*^*B*^ double mutant clones (S3A–S3A” Fig) and *lrpprc2*^*A*^ mutant clones in *MUL1*^*A6*^ mutant background (S3B–S3B” Fig) similar to our observation in *lrpprc2*^*A*^ clones (Fig 3A–3A”). Interestingly, *lrpprc2*^*A*^ mutant clones in *park*^*Δ21*^ mutant background did not show Marf::HA downregulation suggesting Park, and not HUWE1 or MUL1, is required for Marf downregulation in *lrpprc2*^*A*^ (Fig 3B–3B”). Since *park* is known to function downstream to *Pink1* [58,59], and previously a role for PINK1 and Park in Marf downregulation has been established [10], we tested whether PINK1 is also required for Marf degradation in *lrpprc2*^*A*^ mutant clones. We generated *lrpprc2*^*A*^ *Pink1*^*5*^ double mutant clones and found that these clones do not show reduction in Marf::HA levels (Fig 3C–3C”), suggesting that mitochondrial impairment in *lrpprc2* mutant cells could cause PINK1-Park activation and subsequently Marf downregulation. Our observations may relate to reports of downregulation of Mfn1 and Mfn2 upon CCCP treatment as a mechanism to suppress mitochondrial fusion prior to PINK1-Park mediated mitophagy [9,11].

### UPR^mt^ may not be sufficient to induce Marf downregulation

The role of the PINK1-Park pathway in mitochondrial quality control is well known. However, the exact mechanism of PINK1-Park activation in *in vivo* contexts remains unclear. We first checked PINK1 levels in *lrpprc2*^*A*^ mutant clones using a genomically tagged PINK1::Myc line, we found no significant difference in total PINK1::Myc levels between *lrpprc2*^*A*^ mutant clones and neighboring wildtype cells (S3C–S3C” Fig). In cancer cell lines, dissipation of MMP and increased oxidative stress have been shown to activate PINK1-Park on the OMM leading to mitophagy [19,60]. However, we have earlier shown that *lrpprc2* mutants do not have increased oxidative stress as compared to control [39]. We checked MMP in *lrpprc2*^*A*^ mutant clones using TMRE, a dye that reversibly stains mitochondria in a membrane potential-dependent manner. We observed that TMRE intensity in *lrpprc2*^*A*^ mutant clones is similar to that of wildtype cells (S3D–S3D” Fig). These observations rule out the possibility that PINK1-Park is activated due to oxidative stress or altered MMP in *lrpprc2* mutants.

Mitochondrial unfolded protein response (UPR^mt^), which is a cellular response to altered mitochondrial proteostasis, has also been shown to activate PINK1-Park leading to mitophagy [61]. Therefore, we tested for UPR^mt^ activation in *lrpprc2* mutants by checking levels of Hsp60, which is reported to be increased due to elevated UPR^mt^ [62]. We found increased levels of Hsp60A protein in *lrpprc2* mutant clones suggesting elevated UPR^mt^ (S4A–S4A” Fig). Activation of UPR^mt^ upon the loss of *LRPPRC* has also been observed in *C*.*elegans* and mammalian cells and could be evolutionarily conserved [63]. Increased UPR^mt^ could activate PINK1-Park, leading to Marf degradation. We genetically suppressed the UPR^mt^ response pathways and checked its impact on Marf levels in *lrpprc2* mutants. We generated *lrpprc2*^*A*^ mutant clones in the background of *crc*, *foxo* or *dve* knock downs, which are transcription factors mediating UPR^mt^ [64–67]. None of these interventions affected Marf::HA downregulation in *lrpprc2*^*A*^ clones, suggesting that the activation of these UPR^mt^ pathways may not be causing PINK1-Park activation (S4B–S4D” Fig). However, these interventions would not change the altered mitochondrial proteostasis in *lrpprc2* mutants, which can still activate PINK1-Park.

Since, to the best of our knowledge, there is no reported method to suppress mitochondrial proteostasis defects, we asked whether inducing mitochondrial proteostasis defects is sufficient to cause Marf degradation. To induce mitochondrial proteostasis defects, we expressed a mutant form of ornithine transcarbamylase (ΔOTC) that accumulates in an unfolded state in the mitochondrial matrix and is shown to trigger UPR^mt^ in flies [67]. We expressed ΔOTC in the posterior half of the wing disc (marked by RFP) using *En*>*Gal4* (*En*>*Gal4*/+; *UAS-ΔOTC*/+) and found that *ΔOTC* expression shows increased Hsp60 levels indicating UPR^mt^ (Mean increase is 1.166 ± 0.0313 times) (S4E–S4E” Fig). However, the Hsp60 level increase was to a lesser extent compared to *lrpprc2* mutant clones (Mean increase is 1.376 ± 0.02939 times). We checked Marf::HA levels on wildtype *OTC* and *ΔOTC* expression using *En*>*Gal4* and found no change in Marf::HA levels in the posterior half (marked by RFP) as compared to the anterior half of the wing discs for both (S4F–S4G” Fig). Although these observations do not rule out a role for mitochondrial proteostasis in activating PINK1-Park pathway in *lrpprc2* mutants, our data suggest that UPR^mt^ induced by expression of *ΔOTC* is not sufficient to cause Marf degradation.

### Bendless, a K63-linked E2 ubiquitin conjugase, is a regulator of Marf

**Fig 4:**
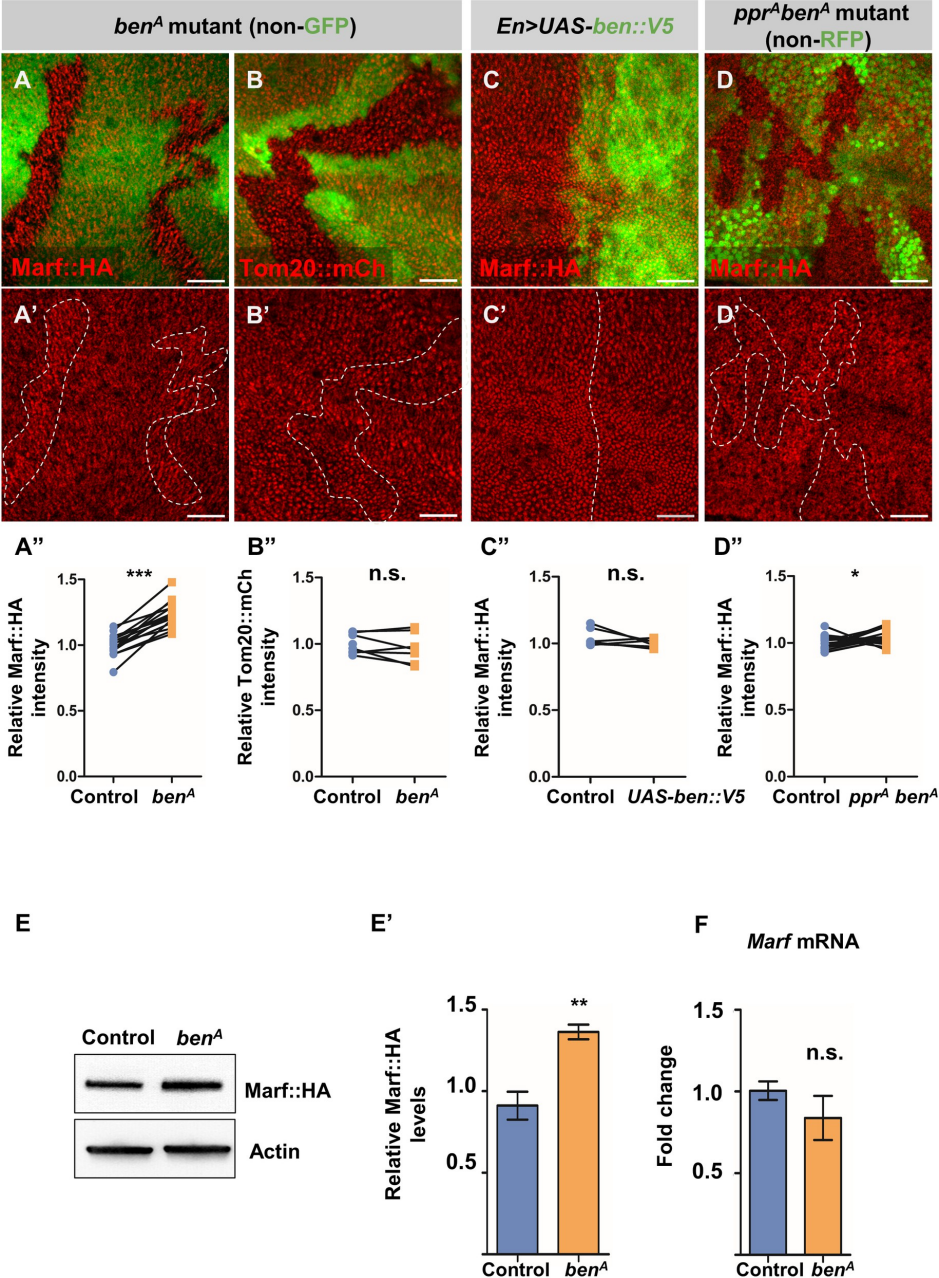
Ben is required for Marf degradation in *lrpprc2* mutants. *(A-B’) ben*^*A*^ mutant clones (non green cells, *A*, *B* and dashed white line, *A’*, *B’*), wing discs immunostained for Marf::HA (red, *A-A’*), Tom20::mCh (red, *B-B’*). *(C-C’)* Overexpression of *ben*::*V5* using *En>Gal4*, wing discs immunostained for Ben::V5 (green) and Marf::HA (red). *(D-D’) lrpprc2*^*A*^ *ben*^*A*^ double mutant clones (non green cells, *D* and dashed white line, *D’*), wing discs immunostained for Marf::HA (red). Scale bar represents 20μm. *(A”*, *B”*, *C”* and *D”)* Quantification for relative fluorescence intensities of Marf::HA in *ben*^*A*^ mutant clones (*A”*, n = 15), Tom20::mCherry in *ben*^*A*^ mutant clones (*B”*, n = 7), Marf::HA levels on Ben::V5 overexpression (*C”*, n = 6) and in *lrpprc2*^*A*^ *ben*^*A*^ double mutant clones (*D”*, n = 16). Graphs represent average intensity values normalized to that of control cells. Two-tailed unpaired t-test between control and mutant cells/cells expressing *UAS-ben*::*V5*. *(E)* Representative western blot for *ben* mutant (*y w ben*^*A*^ FRT19A) and control (*y w* FRT19A) larval lysate probed for Marf::HA and Actin. *(E’)* Quantification for intensity of Marf::HA band normalized to Actin band intensity for *ben*^*A*^ mutant and control larvae (n = 5). Two-tailed unpaired t-test between control and *ben*^*A*^ mutant larvae. *(F)* Quantification of *Marf* mRNA levels in third instar *ben* mutant (*y w ben*^*A*^ FRT19A) larvae compared to control *(y w* FRT19A) (n = 4). Two tailed unpaired *t*-test between control and *ben*^*A*^ mutant larvae. Error bars represent S.E.M. Significance represented by n.s.—non significant, p<0.05*, p<0.01**, p<0.0001***.

In addition to *lrpprc2*, through the genetic mosaic screen we also identified *ben* as a regulator of Marf. We found a subtle but consistent increase in Marf::HA levels in mutant clones of two independent EMS allele of *ben* (*ben*^*A*^ and *ben*^*B*^) (Figs 4A–4A”, S5C–S5C” and S5G), which is similar to that of *park*^*Δ21*^ and *Pink1*^*5*^ mutant clones (S5A–S5B” Fig). We further confirmed increased Marf levels in *ben* mutants by western blot using whole larval extracts (Fig 4E–4E’). In a previous study, *ben* knockdown by RNAi did not alter Marf levels [68] possibly due to inefficient knockdown—the efficacy of RNAi may not be comparable with the two independent loss of function alleles we used. Ben is a fly homologue of the E2 conjugase UBE2N/UBC13 with a marked similarity from yeast to humans (S5E Fig). We ruled out the possibility that the increase in Marf::HA levels upon loss of Ben is due to increased mitochondrial content as there was no difference in Tom20::mCherry levels between *ben* mutant clones and control (Fig 4B–4B”). Further, we did not find an increase in *Marf* mRNA levels in *ben* mutants suggesting that the increase in Marf protein levels is not a consequence of increased transcription (Fig 4F). These data suggest that Ben regulates Marf levels post-transcriptionally.

Next, we asked whether Ben overexpression can induce Marf degradation. To test this, we generated a C-terminal V5-tagged Ben *(UAS-ben*::*V5)* transgenic line for tissue-specific expression of *ben*. We first confirmed that the fusion protein is biologically functional by complementing the lethality associated with the *ben*^*A*^ mutant allele (S5F Fig). We then expressed *ben*::*V5* in the posterior half of the wing disc (marked by green) using the *En*>*Gal4* and found that *ben*::*V5* overexpression did not affect Marf::HA levels (Fig 4C–4C”). Additionally, we overexpressed an N-terminal HA-tagged Ben (*UAS-HA*::*ben*) using *En*>*Gal4* and found no change in Marf::mCherry levels (S6A–S6A” Fig). These data suggest that Ben is necessary but not sufficient for Marf regulation. Since loss of *ben*, *Pink1* or *park* results in mild upregulation of Marf, we hypothesize that Ben acts in the PINK1-Park pathway to regulate the steady state levels of Marf.

### Bendless is essential for Marf downregulation in *lrpprc2* mutants

Given that Marf undergoes proteolytic degradation in *lrpprc2* mutants, we wanted to check if Ben is involved in Marf degradation not only basally but under mitochondrial stress as well, similar to PINK1 and Park. We thus created *lrpprc2* and *ben* double mutant clones and found that *lrpprc2*^*A*^ *ben*^*A*^ and *lrpprc2*^*A*^ *ben*^*B*^ double mutant clones showed no reduction in Marf::HA levels, unlike *lrpprc2*^*A*^ mutant clones (Figs 4D–4D” and S5D–S5D”). This suggests that Ben is essential for Marf degradation in *lrpprc2* mutant cells.

### Ben is required for PINK1 mediated Marf degradation

**Fig 5:**
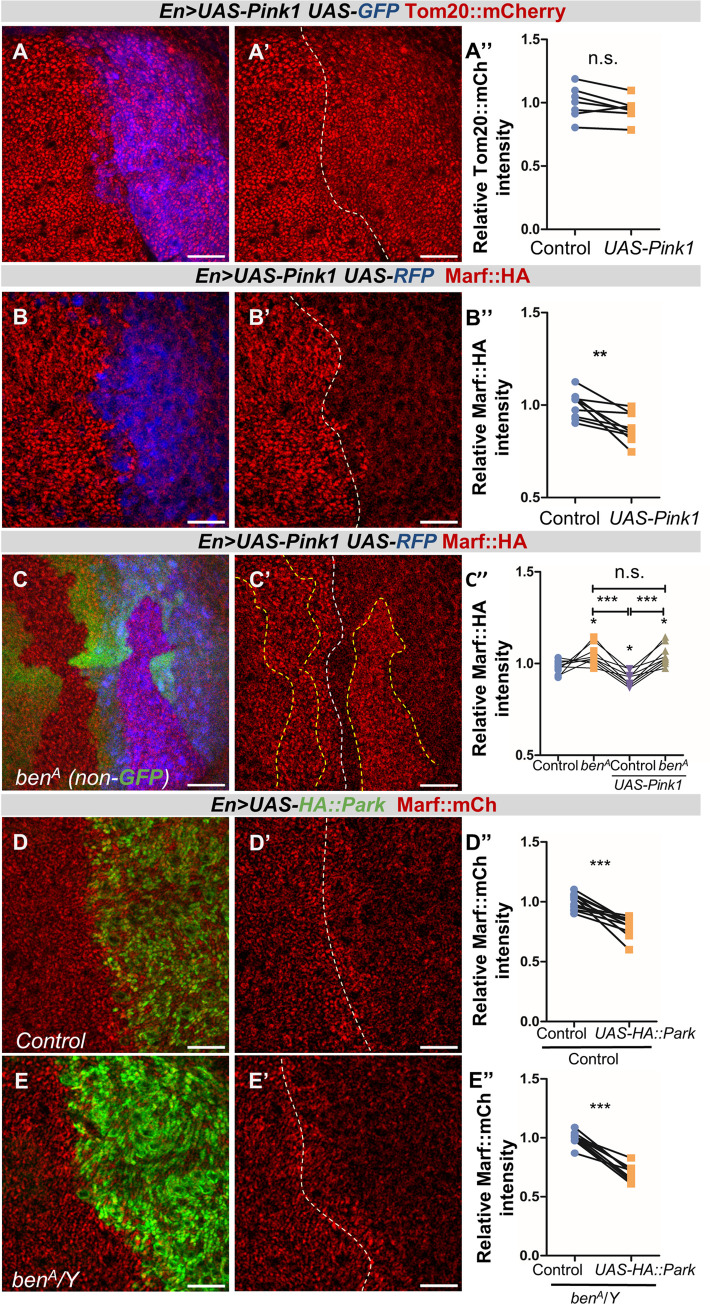
*ben* is required for PINK1 mediated Marf degradation. *(A-A’* and *B-B’)* Overexpression of *Pink1* using *En*>*Gal4*, wing discs marked with *UAS-GFP/RFP* (blue, *A* and *B*) and immunostained for Tom20::mCherry (red, *A-A’*) and Marf::HA (red, *B-B’*). *(C-C’) ben*^*A*^ mutant clones (non green cells, *C* and dashed yellow line, *C’*) in background of overexpression of *Pink1* using *En*>*Gal4*, wing discs marked with *UAS-RFP* (blue, *C*) and immunostained for Marf::HA (red, *C-C’*). *(D-D’ and E-E’)* Overexpression of *Park* using *En*>*Gal4*, wing discs immunostained for HA (green, *D* and *E*) and Marf::mCherry (red) in control (*D-D’*) and *ben*^*A*^ mutant *(E-E’)* wing imaginal discs. Scale bar represents 20μm. *(A”-E”)* Quantification for relative fluorescence intensities of Tom20::mCherry in *UAS-Pink1* cells (*A”*, n = 7), Marf::HA in *UAS-Pink1* cells (*B”*, n = 9), in *ben*^*A*^ mutant clones in wildtype background (*C”*, n = 9), *ben*^*A*^ mutant clones in *UAS-Pink1* background (*C”*, n = 9) and Marf::mCherry in *UAS-HA*::*Park* cells in control (*D”*, n = 12) and *ben*^*A*^ mutant (*E”*, n = 9). Graphs represent average intensity values normalized to that of control cells. Two-tailed unpaired t-test between control and cells overexpressing *UAS-Pink1/UAS-HA*::*Park* in *A”*, *B”*, *D”* and *E”*. A one-way ANOVA-Bonferroni’s multiple comparison test was used to calculate the significance between the samples in graph *C”*. Significance represented by n.s.- non significant, p<0.01**, p<0.0001***.

To study the role of Ben in PINK1-Park mediated regulation of Marf, we tested the functional interaction between *ben* and *Pink1*. Since PINK1-Park activity is suppressed on PINK1 degradation [69], we expected that PINK1 overexpression could activate Marf downregulation in wing discs. Thus, we overexpressed PINK1 in the posterior half (marked by RFP) of the discs (*En*>*Gal4*/*UAS*-*Pink1*, *UAS-RFP* or *UAS-GFP*) and checked the levels of Marf::HA along with other mitochondrial proteins Tom20::mCherry and Complex V. We observed a marked reduction in Marf::HA levels on Pink1 overexpression (Fig 5B–5B”) as compared to Tom20::mCherry and Complex V (Figs 5A–5A” and S7C–S7C”). *Pink1* mutants show increased Marf::HA levels (S5B–S5B” Fig), while overexpression of *Pink1* shows a decrease, it suggests that PINK1 is both necessary and sufficient to downregulate Marf. Further, the decrease in Marf::HA is significantly lower on *Pink1* overexpression as compared to in *lrpprc2*^*A*^ mutant clones (Mean normalized intensity of *lrpprc2*^*A*^ and *Pink1* overexpression are 0.7581 ± 0.02080 and 0.5796 ± 0.05410 respectively) and hence overexpression of *Pink1* could mask the downregulation observed in *lrpprc2* mutants (S7E–S7F Fig). Interestingly, *Pink1* overexpression in the wing discs may not have a significant impact on mitophagy as the mitochondrial content, (Tom20::mCherry and Complex V levels, Figs 5A–5A’ and S7C–S7C”) is not affected.

To test the functional interaction between Ben and PINK1, we created *ben*^*A*^ mutant clones in both wildtype and *Pink1* overexpression backgrounds in the same imaginal discs and found that *Pink1* overexpression does not induce Marf::HA downregulation in *ben*^*A*^ mutant clones (Fig 5C–5C”). Similarly, overexpression of PINK1 in *ben*^*A*^ mutant wing discs does not cause Marf downregulation (S7A–S7A’ Fig). These data suggest that Ben is essential for PINK1 mediated Marf reduction.

Further, we tested the effect of *Park* overexpression on Marf levels. We found overexpression of HA tagged *Park (En>Gal4/UAS-HA*::*Park)* results in reduced Marf::mCherry levels (Fig 5D–5D”), while Complex V levels remain unaltered (S7D–S7D” Fig). We then overexpressed *Park* in *ben*^*A*^ mutant discs—interestingly we found loss of *ben* could not suppress reduction of Marf::mCherry due to *Park* overexpression (Fig 5E–5E”) suggesting that overexpression of *Park* can override loss of *ben*. Overall, these experiments suggest that Ben is essential for PINK1 mediated regulation of Marf and acts genetically upstream to Park.

### Ben regulates PINK1 stability

**Fig 6:**
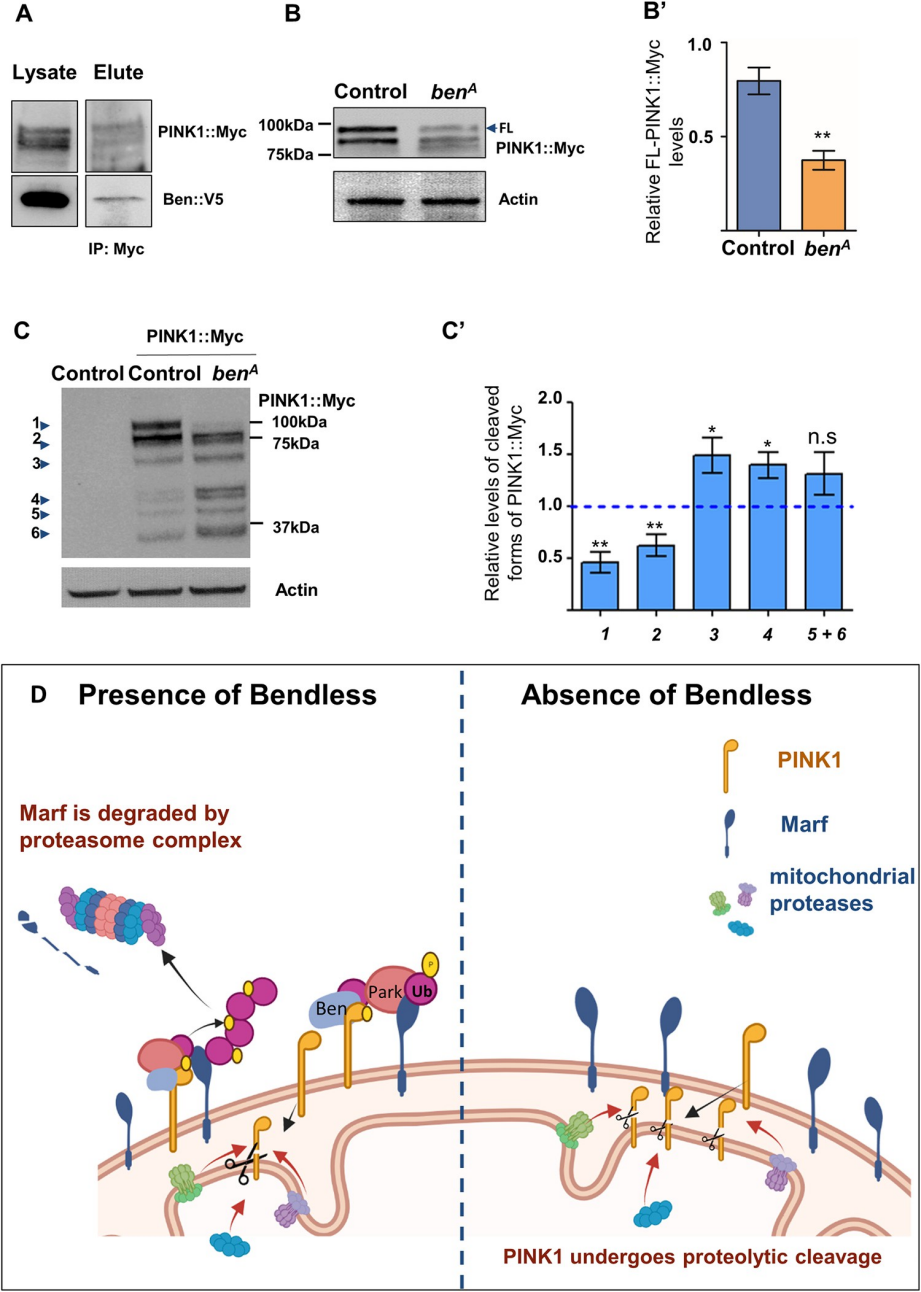
Ben regulates PINK1 stability. *(A)* Representative western blot of PINK1::Myc immunoprecipitation followed by staining for PINK1::Myc and Ben::V5 *(B)* Representative western blot for *ben* mutant (*y w ben*^*A*^ FRT19A; *Pink1*::*Myc*) and control (*y w* FRT19A; *Pink1*::*Myc*) larval lysate probed for PINK1::Myc and Actin. *(B’)* Quantification for intensity of full length (FL) PINK1::Myc band normalized to Actin band intensity for *ben*^*A*^ mutant and control larvae (n = 8). Two-tailed unpaired t-test between control and mutant larvae. *(C)* Representative western blots for control (*y w* FRT19A), control with PINK1::Myc (*y w* FRT19A; *Pink1*::*Myc*) and *ben* mutant (*y w ben*^*A*^ FRT19A; *Pink1*::*Myc*) larval lysate probed for Myc and Actin. *(C’) Ratio of PINK1*::*Myc bands of ben*^*A*^ mutant and control with PINK1::Myc larvae (n = 8). *PINK1*::*Myc band was normalized to Actin*. One sample two-tailed t-test of the ratios of PINK1::Myc bands was done Theoretical mean, 1.0, represented as blue dashed lines. Same samples were used to quantify results as in Fig 6B. Error bars represent S.E.M. Significance represented by p<0.05*, p<0.01**, p<0.0001***, n.s—non significant. *(D)* Schematic depicting Ben-PINK1 regulation and Ben-PINK1-Parkin mediated steady-state turnover of Marf. Created using Biorender.com.

To understand how Ben regulates PINK1, we first checked Ben and PINK1 protein interaction using co-immunoprecipitation. We used *the UAS-Ben*::*V5* and genomically tagged PINK1::Myc fly lines (*w;PINK1*::*Myc/+;Actin>Gal4/UAS-ben*::*V5*) and pulled down PINK1:Myc. As shown in Fig 6A, probing for Ben::V5 on pull down of PINK1::Myc shows presence of Ben::V5 indicating Ben and PINK1 directly interact. Further, we checked the effect of loss of *ben* on PINK1 levels. We performed western blots using whole larval extracts from control and *ben*^*A*^ mutants containing genomic tagged PINK1::Myc. We found a significant downregulation of full length PINK1::Myc in *ben*^*A*^ mutants, but an increase in low molecular weight PINK1::Myc bands (Fig 6B–6C’). This suggests that Ben is required for stabilizing full length PINK1. The low molecular weight bands might be products of PINK1 degradation by mitochondrial proteases as described by Thomas et.al. [70]. Taken together, our data suggests that Ben is required for the stability of PINK1 which mediates the homeostatic turnover of Marf (Fig 6D).

### Ben regulates mitochondrial dynamics under mitochondrial stress

**Fig 7:**
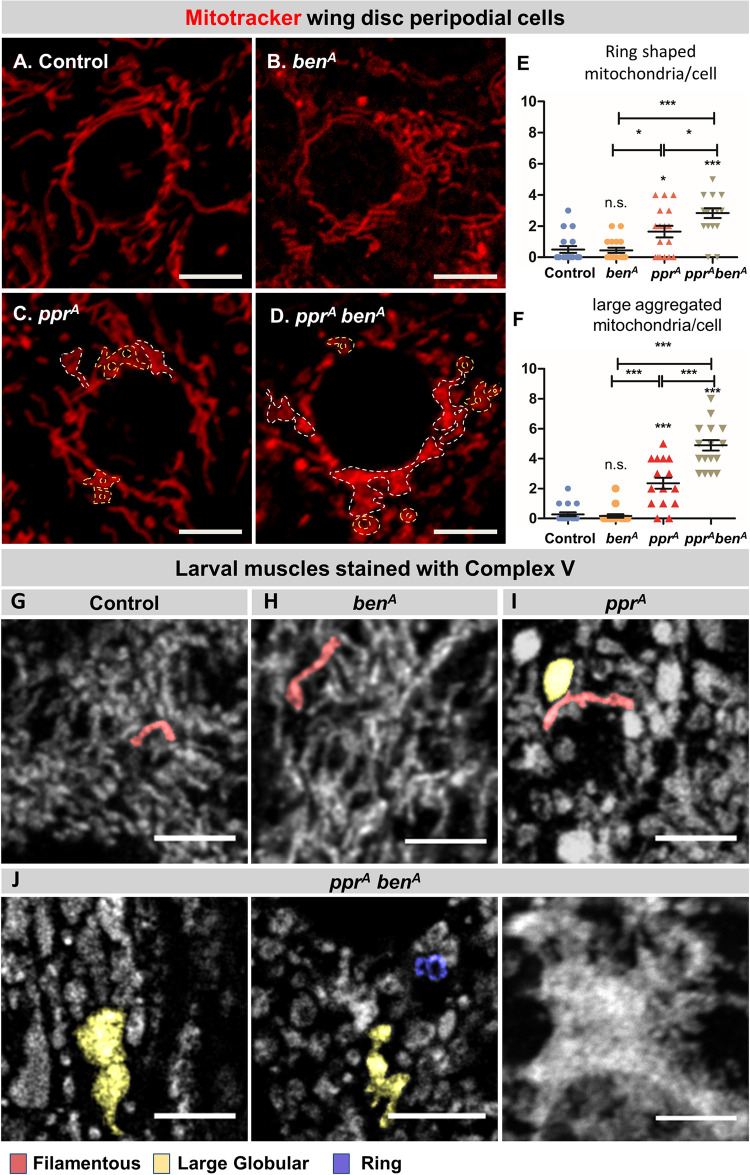
Ben is required for maintaining mitochondrial morphology in *lrpprc2* mutants. *(A-D)* Wing disc stained for Mitotracker Red (red) and imaged live for control*(A)*, *ben*^*A*^*(B)*, *lrpprc2*^*A*^*(C)* and *lrpprc2*^*A*^ *ben*^*A*^*(D)* mutant clones in peripodial cells. White dashed lines mark aggregated mitochondria and yellow dashed lines mark ring shaped mitochondria. Scale bar represents 5μm. *(E)* Dot plot representing the number of ring shaped mitochondria present in one peripodial cell, the center line represents the mean value (n = 18). *(F)* Dot plot representing the number of large aggregated mitochondria present in one peripodial cell, the center line represents the mean value (n = 18). Error bars represent S.E.M. A one-way ANOVA-Tukey’s multiple comparison test was used to calculate the significance between the samples in graph *(E)* and *(F)*. Significance represented by n.s—non significant, p<0.05*, p<0.01**, p<0.0001*** *(G-J)* Confocal sections of third instar larval muscles immunostained for Complex V (gray) in control*(G)*, *ben*^*A*^*(H)*, *lrpprc2*^*A*^*(I)* and *lrpprc2*^*A*^ *ben*^*A*^*(J)* larvae. Representative individual mitochondrial morphology is marked by different colors: filamentous (red), large globular (yellow) and ring (blue). Scale bar represents 5μm.

Given the role of Ben in Marf regulation under steady state conditions as well as in *lrpprc2* mutants, we sought to investigate and compare mitochondrial morphology between control, *ben*, *lrpprc2* and *lrpprc2 ben* double mutants. First, we compared mitochondrial morphology in mutant clones in peripodial cells of wing discs using mitotracker red staining and live imaging (Fig 7A–7D). We found that the mitochondrial morphology in *ben* mutant cells is comparable to wildtype cells—they both show a filamentous network of mitochondria. A previous study, however, has shown increased mitochondrial size due to *ben* knockdown in the fat body—the difference in the phenotype could be due to tissue specific differences in mitochondrial physiology [71]. In *lrpprc2*^*A*^ mutant cells, we observe filamentous mitochondria along with large aggregated mitochondria and ring-shaped mitochondria (Fig 7A–7F). Further, we found that the large aggregated mitochondria and ring-shaped mitochondrial phenotype are worsened in *lrpprc2*^*A*^ *ben*^*A*^ double mutant cells (Fig 7C–7F). Compared to fixed samples (S1D–S1D” Fig), live imaging showed more tubular and networked mitochondria (Fig 7A–7D). This difference in mitochondrial morphologies could be owing to the difference in sample preparations as also documented earlier [72]. However, in both scenarios *lrpprc2*^*A*^ mutants consistently show presence of large mitochondria as compared to wildtype cells. We further investigated mitochondrial morphology in larval muscles using Complex V antibody staining and we found wildtype and *ben*^*A*^ mutants show a comparable filamentous network of mitochondria (Figs 7G–7H and S8A–S8B); *lrpprc2* mutants show distinctive large globular mitochondria along with filamentous and ring shaped mitochondria (Figs 7I and S8C); *lrpprc2 ben* double mutants rarely show filamentous mitochondria, instead, we observed a significant increase in the size and frequency of large globular and ring-shaped mitochondria as compared to *lrpprc2* (Figs 7J and S8D). We also observed that in lesser frequency mitochondria in *lrpprc2*^*A*^ *ben*^*A*^ double mutants form clusters, especially around the nucleus which is not observed in either *lrpprc2*^*A*^ or *ben*^*A*^ mutants (S8D Fig). To report these mixed phenotypes we have documented several images for each genotype in Figs 7 and S8.

**Fig 8:**
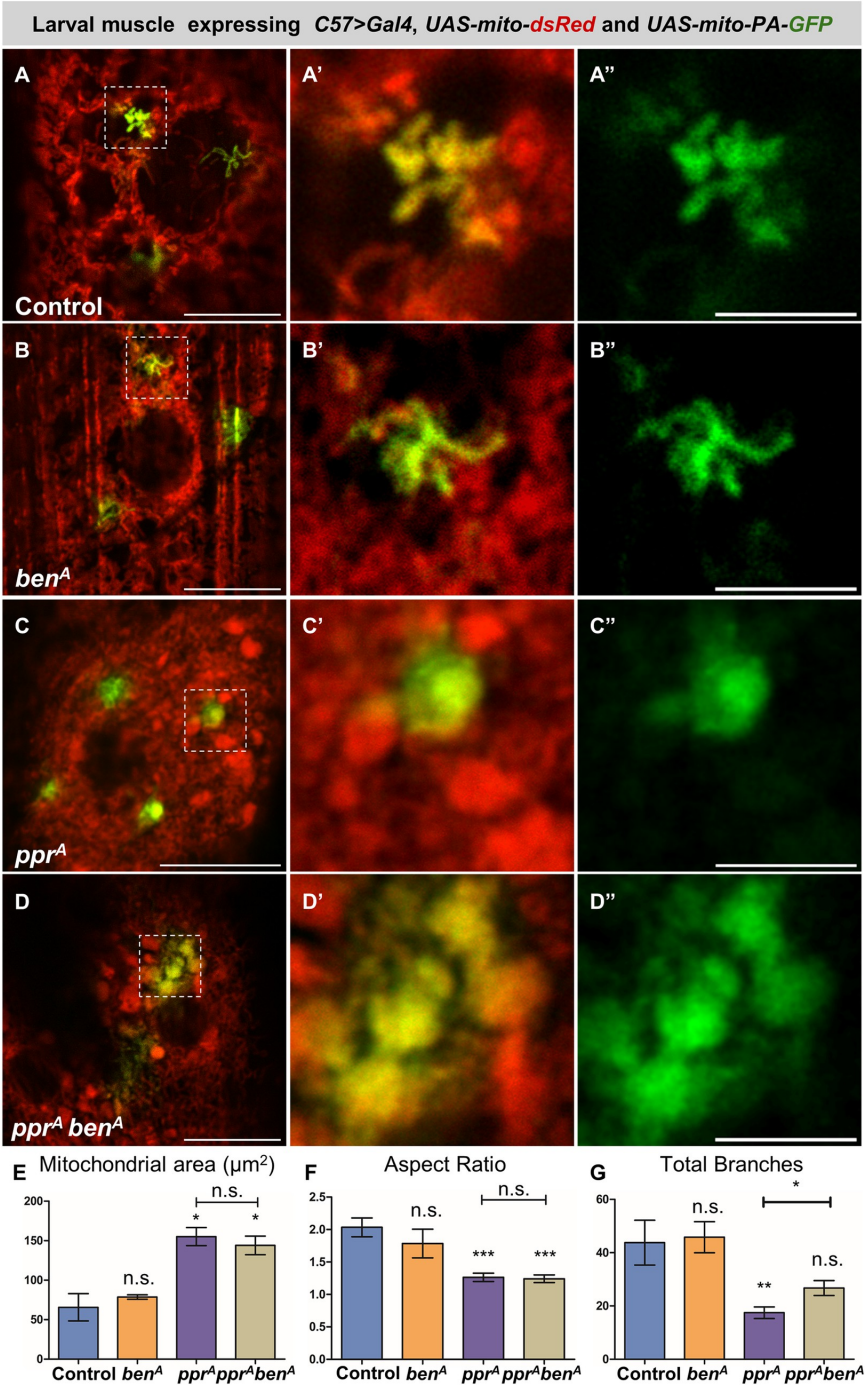
Ben limits mitochondrial branching in *lrpprc2* mutants. *(A-D”)* Third instar larval muscles expressing *UAS-mito-dsRed* (red) and *UAS-mito-PA-GFP* (green marks photoactivated mitochondrial regions) under *C57>Gal4* in control*(A)*, *ben*^*A*^*(B)*, *lrpprc2*^*A*^*(C)* and *lrpprc2*^*A*^ *ben*^*A*^*(D)* larvae. Yellow dashed lines mark the insets. Scale bar represents 15μm in *A-D* and 5μm for insets. *(E)* Bar graph representing average area of individual mitochondria. *(F)* Bar graph representing average aspect ratio of mitochondria. *(G)* Bar graph representing total branch number. Error bars represent S.E.M. A one-way ANOVA-Tukey’s multiple comparison test was used to calculate the significance between the samples in graph *(E-G)*. Significance represented by n.s—non significant, p<0.05*, p<0.01**, p<0.0001***.

**Fig 9:**
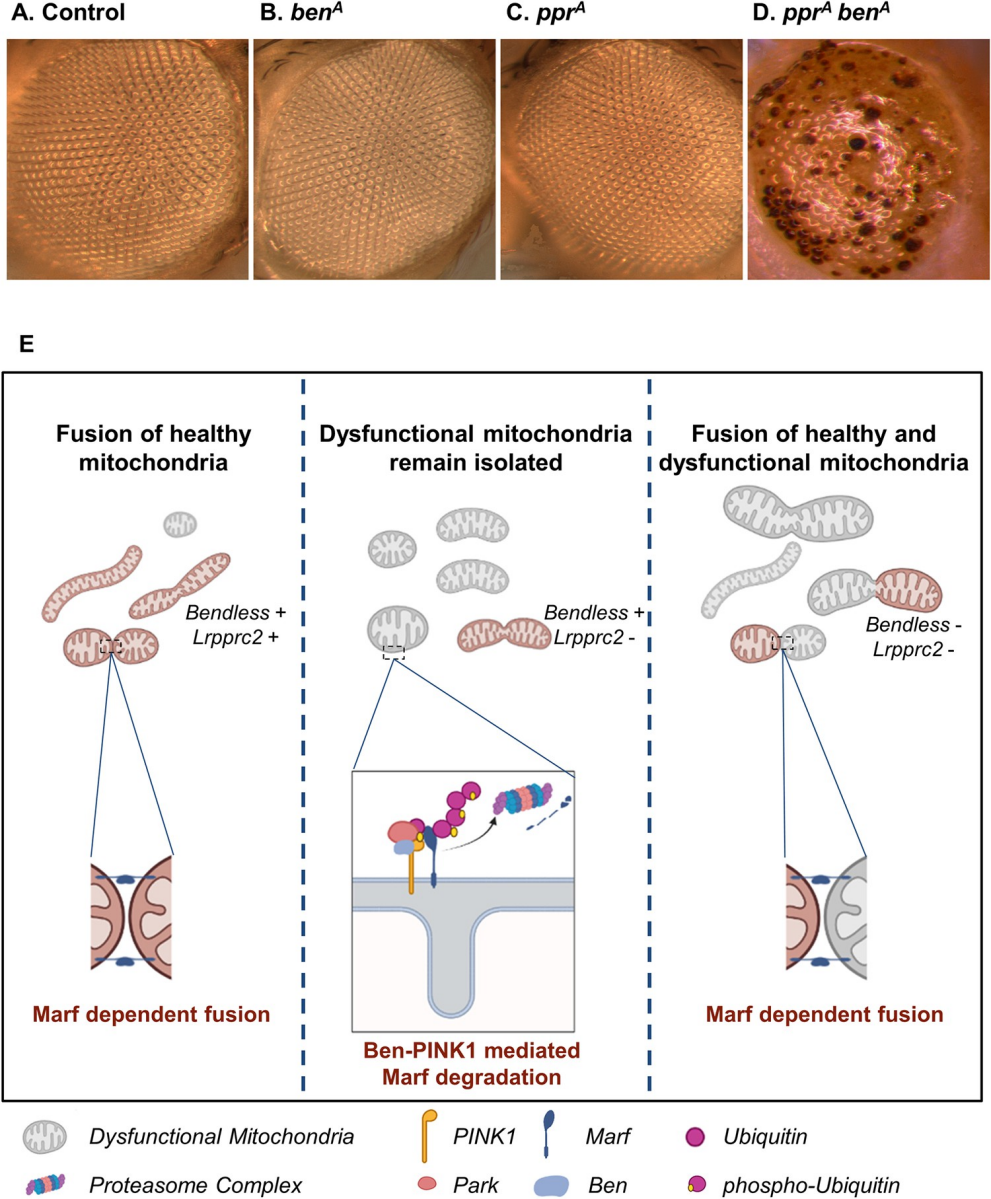
Loss of Ben enhances eye degeneration in *lrpprc2* mutants. *(A-D)* Mutant eye clones from young flies of control*(A)*, *ben*^*A*^*(B)*, *lrpprc2*^*A*^*(C)*, and *lrpprc2*^*A*^ *ben*^*A*^*(D)* genotypes. The images within the figure panels are created by the authors. *(E)* Schematic representing Ben-PINK1 mediated mitochondrial size control in *lrpprc2* mutants. Loss of *lrpprc2* results in mitochondrial dysfunction (gray) which causes Ben-PINK1 activation to suppress mitochondrial fusion between healthy and dysfunctional mitochondria. In the absence of Ben mitochondrial segregation is lost giving rise to aberrant mitochondria. Created using Biorender.com.

To further resolve the mitochondrial morphology and quantify various features of individual mitochondria in larval muscles we used mitochondrial photoactivatable GFP (Mito-PA-GFP). This allows visualization of individual mitochondria and its network within a cell by activating GFP fluorescence, using 405 nm laser, in a region of interest [73,74]. We found a comparable filamentous network of mitochondria in control and *ben*^*A*^ mutants as they show similar branch numbers and aspect ratios (Fig 8A–8B” and 8E–8G). In *lrpprc2*^*A*^ mutants we observed globular shaped mitochondria characterized by larger area and lower aspect ratio. *lrpprc2*^*A*^ mutants also show marked reduction in mitochondrial network as characterized by reduced branch number (Fig 8C–8C” and 8E–8G). In *lrpprc2*^*A*^ *ben*^*A*^ double mutants we observed the presence of globular shaped mitochondria characterized by larger area and lower aspect ratio (Fig 8D–8D” and 8E–8G). As compared to *lrpprc2*^*A*^, in which globular mitochondria remain isolated, in *lrpprc2*^*A*^ *ben*^*A*^ we observed globular mitochondria are interconnected (Fig 8D–8D”). This is also reflected in the increase in branch numbers in the case of *lrpprc2*^*A*^ *ben*^*A*^ double mutants when compared to *lrpprc2*^*A*^ mutants (Fig 8G). Overall, our results suggest that mitochondrial dysfunction in *lrpprc2* may induce the formation of globular mitochondria and Ben mediated regulation of Marf suppresses their fusion (Fig 9E).

### Accelerated retinal degeneration and abnormality in wing patterning in *lrpprc2 ben* double mutants

Mutations in human *LRPPRC* cause Leigh Syndrome, a neurometabolic disease [40] and loss of *lrpprc2* in *Drosophila* causes activity induced retinal degeneration [39]. As *lrpprc2 ben* double mutants exacerbate the mitochondrial morphology phenotypes, we suspected that the loss of *ben* may enhance retinal degeneration. To test this, we made eye specific *ben*^*A*^, *lrpprc2*^*A*^ and *lrpprc2*^*A*^ *ben*^*A*^ double mutant clones using the *ey-FLP* system [44]. We found that *lrpprc2* mutant and *ben*^*A*^ mutant eyes show normal morphology upon eclosion. However, *lrpprc2*^*A*^ *ben*^*A*^ double mutant eyes show severe retinal degeneration suggesting that loss of *ben* could accelerate retinal degeneration in *lrpprc2*^*A*^ (Fig 9A–9D). This suggests that Marf regulation by Ben is a neuroprotective mechanism. Additionally, we also investigated adult wing phenotype in *lrpprc2 ben* double mutants. We used *Ubx-FLP* to generate large clones in the developing wing. Most of the wings in *lrpprc2*^*A*^ and *ben*^*A*^ single mutants were normal, with occasional minor defects in bristle pattern in both mutants (S9A–S9C Fig). However, in *lrpprc2*^*A*^ *ben*^*A*^ double mutants we see the following: most flies eclose with improperly folded wings, additionally these mutant wings show wing patterning defects such as presence of ectopic veins and dark patches on the wing blade (S9D Fig). Together these data suggest a protective role of Bendless upon mitochondrial stress and this becomes prominent in the case of neurons, possibly due to their high energy requirements.

## Discussion

To identify novel regulators of mitochondrial fusion in an *in vivo* system, we screened fly mutants for altered Marf levels and identified mutations in *lrpprc2* causing reduction in Marf levels (Fig 1A). We found that in *lrpprc2* mutants, Marf is degraded by the UPS (Fig 2C–2D) in a PINK1-Park dependent mechanism (Fig 3A–3C). In the screen, we also identified mutations in the E2 conjugase *ben*, causing subtle Marf upregulation (Fig 4A). We found that Ben is essential for PINK1 stability **(**Fig 6B), regulates Marf levels (Fig 4D) and mitochondrial morphology (Figs 7D, 7J and 8D) in *lrpprc2* mutants. We also found that a combined loss-of-function mutation of *lrpprc2* and *ben* in the eyes results in accelerated retinal degeneration (Fig 9D) and developmental abnormalities in wings (S9D Fig). Indicating that under mitochondrial stress induced by loss of *lrpprc2*, Ben mediated regulation of mitochondrial dynamics is a protective response (Fig 9E).

Increased mitochondrial size and globular mitochondrial phenotype, as observed in *lrpprc2* mutants (Figs S1E, 7C and 8E), have been observed in certain metabolic diseases [75,76]. Increased mitochondrial size has also been observed upon loss of *lrpprc2* homologs in *C*.*elegans*, mouse and human cell lines [42,43] as well as in other mutants where the ETC is compromised [62,77,78]. The mechanism of such responses, and how these unusual shaped mitochondria contribute to cell physiology and disease progression, is not clear. As SIMH has been observed in a bid to increase oxidative phosphorylation under various cellular and mitochondrial stresses [5,79,80], we hypothesize that reduced ETC activity and mitochondrial stress in *lrpprc2* [38,39,43] can induce mitochondrial enlargement (Figs 6D and S1E), similar to SIMH, through an unknown mechanism. Since SIMH increases ATP synthesis and inhibits mitophagy [3,5,81,82], increased mitochondrial size could be a compensatory adaptation in *lrpprc2* mutants in response to a bioenergetic deficit or mitochondrial stress. Further, globular shaped mitochondria could also be a favorable adaptation as it is recently shown that, in comparison to elongated mitochondria, globular mitochondria contain densely packed cristae membranes with high curvature. As ATP synthase is known to localize at intense curvature in cristae, globular mitochondria might possess better energetic capabilities [83,84]. Further ultrastructural analysis along with mitochondrial activity assays may shed light on this possibility.

Despite the increased mitochondrial size (S1E Fig), we observed Marf downregulation in *lrpprc2* mutants (Fig 1A). We hypothesize that, while an adaptive mechanism may induce SIMH *(cellular response)*, MQC may induce Marf degradation to suppress the fusion of dysfunctional mitochondria (*mitochondrial response*) (Fig 8D). Indeed we observed that Marf reduction in *lrpprc2* mutants is correlated with the presence of large globular mitochondria that remain isolated (Fig 8E–8G). A similar scenario of isolation of dysfunctional mitochondria prior to mitophagy has been proposed earlier [9–11]. Alternatively, increased mitochondrial size in *lrpprc2* mutant cells may induce the PINK1-Park pathway to limit mitochondrial fusion by Marf degradation. A similar hypothesis was also proposed by Yamada et al. wherein loss of *Drp1* results in Parkin dependent Mitofusin downregulation [85].

We found that Marf degradation in *lrpprc2* mutant clones in developing wing primordium is dependent on Park (Fig 3B). We also observed a subtle increase in Marf levels in *park* and *Pink1* mutant clones (S5A–S5B Fig) (also see [37]). This suggests that PINK1-Park play a homeostatic role in Marf turnover in wildtype tissue, while mitochondrial impairments—as in *lrpprc2* mutants [39]—may further amplify its activity to reduce Marf levels (Fig 1A) possibly to segregate damaged mitochondria [86]. We also find that PINK1 or Park overexpression is sufficient to induce Marf degradation without triggering mitophagy (Figs 5B, 5D, S7C and S7D). *In vivo* studies have shown PINK1-Park to function both in mitophagy [22–29] and mitochondrial dynamics [31–35], but the physiological or cellular contexts that may determine various downstream activities of PINK1-Park are not known [30,87,88]. In *lrpprc2* mutants we observe PINK1-Park mediated Marf degradation in the absence of mitophagy. Hence, *lrpprc2* mutants could provide a novel and physiologically relevant *in vivo* system to study PINK1-Park mediated Marf regulation under mitochondrial stress.

In steady state conditions, PINK1 is imported into the mitochondria and cleaved by mitochondrial peptidases, it then retro translocates to the cytoplasm and is degraded by UPS to limit PINK1-Park activity [70,89,90]. The initial remarkable discovery by Narendra et al. that CCCP which dissipates MMP, induces PINK1-Park-dependent mitophagy in cancer cells provided an unparalleled assay to investigate the mechanism further [19,91]. Further studies also show that increased oxidative stress or UPR^mt^ stabilizes full-length PINK1, which then recruits Park leading to ubiquitination of OMM proteins and mitophagy [10,19,61,92,93]. Given no change in MMP (S3D Fig) and oxidative stress in *lrpprc2* mutants [38,39,49], we suspected that impaired mitochondrial proteostasis activates PINK1-Park to downregulate Marf. However, activation of UPR^mt^ by *ΔOTC* expression did not result in Marf degradation suggesting that activation of UPR^mt^ alone may not be sufficient to activate PINK1-Park mediated Marf degradation *in vivo* (S4G Fig). Identification of the nature of the mitochondrial stressors leading to PINK1-Park activation in *lrpprc2* will require further investigation.

Several regulators of PINK1 stability and activity have been identified. For example, CHIP-mediated K48-ubiquitination promotes PINK1 turnover [94], while BAG2, a chaperon, prevents ubiquitination and promotes PINK1 stability [95,96]. We found that Marf degradation in *lrpprc2* mutants or by *Pink1* overexpression is completely suppressed in the absence of the K63-linked E2 conjugase Ben (Figs 4D, 5C and S5D). However, *park* overexpression could cause Marf downregulation even in the absence of *ben* (Fig 5E). This suggests that Ben regulates PINK1 mediated Marf degradation and that Park may not be directly regulated by Ben. Previous studies have observed that the mammalian homolog of Ben, UBE2N, is dispensable for mitophagy but facilitates the clustering of mitochondria during CCCP-induced mitophagy [68,97,98]. We also found that the loss of *ben* does not alter developmental mitophagy during larval midgut remodeling (S7B Fig), which has been shown to be dependent on PINK1-Park [22,23].

K63 ubiquitination of PINK1 by the Traf6-SARM1 complex is shown to stabilize PINK1 on depolarized mitochondria in mammalian cells [99]. As Ben protein interacts with PINK1 (Fig 6A) and loss of *ben* results in reduced PINK1 levels (Fig 6B), Ben is likely to increase the stability of PINK1 by K63 ubiquitination. Indeed, human PINK1, in cell culture systems, is known to be ubiquitinated at K137 by both K48 and K63 linkages [100]. While K48 chains are linked with PINK1 degradation; the significance of the K63 linkage is not obvious. K63 ubiquitination is suggested to protect proteins from proteasomal degradation [101]. Overall, Ben-mediated K63 ubiquitination could be responsible for PINK1 stability and remains to be tested. We hypothesize that absence of Ben could lead to increased import of PINK1 into the mitochondria hence reducing its full-length levels, even on overexpression of *Pink1*. A similar observation was made by Sekine et. al. with reference to Tom7 [102]. Wherein loss of Tom7, a component of the TOMM complex, resulted in mitochondrial import of PINK1 and cleavage by OMA1 [102]. Additionally, whether Ben regulates PINK1 activity needs further study.

Ben-PINK1-Park regulation of Marf appears to be a homeostatic function which is further activated in response to aberrant mitochondrial function. Compared to *lrpprc2*, *lrpprc2 ben* mutants show increased number of ring shaped, globular and large aggregated mitochondria. We also observed that large globular mitochondria are interconnected in *lrpprc2 ben* double mutants whereas globular mitochondria remain isolated in *lrpprc2* mutants, possibly due to the reduction of Marf. This Ben-PINK1-Parkin mediated Marf degradation in *lrpprc2* appears to be a cell protective mechanism as *lrpprc2 ben* double mutants show accelerated retinal degeneration and worsened adult wing phenotype as compared to *lrpprc2* mutant (Figs 9 and S9). Given that mutations in *LRPPRC* result in Leigh syndrome, it is likely that Ben/Ubc13-PINK1-Park may regulate Mfn1 and Mfn2 in Leigh syndrome as well in other mitochondrial diseases. Indeed, altered mitochondrial dynamics has been reported in many mitochondrial diseases [76,103–105]. Thus, further studies on the mechanisms of Ben/Ubc13-PINK1-Park activation will be crucial for understanding mitochondrial quality control in mitochondrial disease.

## Material and methods

### *Drosophila* culture

Flies were cultured on standard media containing sucrose, malt, yeast and corn flour at room temperature. Crosses were maintained at 25°C. Crosses involving RNAi were maintained at 28°C. *Drosophila* larvae expressing *UAS-Prosβ6*^*1*^ were maintained at 25°C till 3^rd^ instar stage and were then transferred to 28°C for 24 hours before dissection, to avoid cell death observed on prolonged inhibition of proteasomal activity. To activate the *FLP*-FRT system, heat shock was given during first instar larval stages at 37°C for 1hr. Genotypes used are as listed in Table 1.

**Table 1:** *Drosophila* genotypes used in the study.

Genotype	Source
**Fig 1**	
*y*^*1*^ *lrpprc2* ^*A*^ w* *P{neoFRT}19A*	FBst0067166[39],[44]
*Marf*::*HA* (Genomic tag on Chromosome III)	[46]
*Ubiquitin>GFP hsFLP [122]*, *P{neoFRT}19A*	Hugo Bellen
*Ubiquitin>GFP*, *hsFLP [122]*, *P{neoFRT}19A**;; Marf*::*HA*(III)	This study
*Tom20-mCherry* (Genomic construct Chromosome III)	[109]
*Ubiquitin>GFP*, *hsFLP [122]*, *P{neoFRT}19A**;; Tom20-mCherry*	This study
*w;; pacman Opa1*::*HA*::*Flag VK31* (III) *(Opa1*::*HA)*	This study
*Ubiquitin>GFP*, *hsFLP [122]*, *P{neoFRT}19A**;; Opa1*::*HA*::*Flag VK31*	This study
*y*^*1*^ w*; P{w[+mC] = FLAG-FlAsH-HA-Drp1}3, Ki[1]	FBst0042208 [110]
*Ubiquitin>GFP*, *hsFLP [122]*, *P{neoFRT}19A**;; P{w[+mC] = FLAG-FlAsH-HA-Drp1}3, Ki[1]*	This study
**Fig 2**	
*y*^*1*^ *lrpprc2* ^*A*^ w* *P{neoFRT}19A*	FBst0067166 [39],[44]
*Ubiquitin>GFP*, *hsFLP [122]*, *P{neoFRT}19A**;; Marf*::*HA(III)*	This study
*w*;; *Actin*>*Gal4*	Hugo Bellen
*w*;; *Actin>Gal4*, *Marf*::*HA* (III)	This study
*y*^*1*^ *lrpprc2* ^*A*^ w* *P{neoFRT}19A*;; *Actin*>*Gal4*, *Marf*::*HA* (III)	This study
w*; *P{UAS-Prosbeta6[1].B}2B* (II)	FBst0006786 [54]
*Ubiquitin>GFP*, *hsFLP* *P{neoFRT}19A*; *P{UAS-Prosbeta6[1].B}2B* (II)	This study
**Fig 3**	
*y*^*1*^ *lrpprc2* ^*A*^ w* *P{neoFRT}19A*	FBst0067166 [39],[44]
w* Pink1 ^*5*^	FBst0051649 [58]
*y*^*1*^ *lrpprc2* ^*A*^ *Pink1*^*5*^ *P{neoFRT}19A*	This study
w*;; park^*Δ21*^ *(III)*	FBst0051652 [111]
*y*^*1*^ *lrpprc2* ^*A*^ w* *P{neoFRT}19A*;; *park*^*Δ21*^ *Marf*::*HA*	This study
*Ubiquitin>GFP*, *hsFLP [122]*, *P{neoFRT}19A**;; park*^*Δ21*^	This study
w*;P{w[+mC] = Marf-gHA}2 (Genomic construct Chromosome II)	FBst0067156 [46]
*Ubiquitin>GFP*, *hsFLP* [122], *P{neoFRT}19A*; *P{Marf*::*HA}2*	This study
**Fig 4**	
*y*^*1*^ w^*^ *ben*^*A*^ *P{neoFRT}19A*	FBst0057057 [44]
*Ubiquitin>GFP*, *hsFLP [122]*, *P{neoFRT}19A**;; Marf*::*HA* (III)	This study
*Ubiquitin>GFP*, *hsFLP [122]*, *P{neoFRT}19A**;; Tom20*::*mCherry* (III)	This study
*y*^*1*^ w*; *P{en2*.*4-GAL4}e16E*	FBst0030564
*w*; P{*Marf*::*HA*}2 *P{w[+mW*.*hs] = en2*.*4-GAL4}e16E*, *P{w[+mC] = UAS-RFP*.*W}2*	This study
w^*^;;P{UAS.*ben*::*V5*.*w+mC}*	This study
*y*^*1*^ *lrpprc2* ^*A*^ w* *P{neoFRT}19A*	FBst0067166 [39],[44]
*y*^*1*^ *lrpprc2* ^*A*^ w^*^ *ben*^*A*^ *P{neoFRT}19A*	This study
**Fig 5**	
w*;P{w[+mC] = Marf-gHA}2 (Genomic construct Chromosome II)	FBst0067156 [46]
*w*; *P{Marf*::*HA}2* *P{w[+mW*.*hs] = en2*.*4-GAL4}e16E*, *P{w[+mC] = UAS-RFP*.*W}2*	This study
w^*^; *P{w[+mC] = UAS-Pink1*.*C}A*	FBst0051648 [58]
*Tom20-mCherry* (Genomic construct Chromosome III)	[109]
*w; P{w[+mW*.*hs] = en2*.*4-GAL4}e16E*, *P{w[+mC] = UAS-GFP*.*W}2*	Hugo Bellen
*w; P{w[+mW*.*hs] = en2*.*4-GAL4}e16E*, *P{w[+mC] = UAS-GFP*.*W}2; Tom20-mCherry* (Genomic construct Chromosome III)	This study
*y*^*1*^ w^*^ *ben*^*A*^ *P{neoFRT}19A*; *P{Marf*::*HA}2* *P{w[+mW*.*hs] = en2*.*4-GAL4}e16E*, *P{w[+mC] = UAS-RFP*.*W}2*	This study
*Ubiquitin>GFP*, *hsFLP [122]*,*P{neoFRT}19A**;;P{w[+mC] = UAS-Pink1*.*C}A*	This study
*Ubiquitin>GFP*, *hsFLP [122]*,*P{neoFRT}19A*	Hugo Bellen
*y1* w*; *P{en2*.*4-GAL4}e16E*	FBst0030564
*Marf*::*mCherry* (Genomic rescue construct, Chromosome III)	Hugo Bellen
*w*;;*P{UAS-HA-park*.*Y}*	FBal0301084 [57]
*Ubiquitin>GFP*, *hsFLP [122]*,*P{neoFRT}19A*; *P{en2*.*4-GAL4}e16E**; P{UAS-park*.*8HA}*	This Study
*y*^*1*^ w^*^ *ben*^*A*^ *P{neoFRT}19A**;;Marf*::*mCherry (Genomic rescue construct*, *Chromosome III)*	This Study
**Fig 6**	
*P*{*Pink1-9Myc*} (Pink1::Myc) (II)	FBtp0022940 [58]
*y*^*1*^ w^*^ *ben*^*A*^ *P{neoFRT}19A**; P*{*Pink1-9Myc*}	This study
*y*^*1*^ w* *P{neoFRT}19A**; P*{*Pink1-9Myc*}	This study
*w;P*{*Pink1-9Myc*}*; Actin>Gal4*	This study
w*;;P{UAS.*ben*::*V5*.*w+mC}*	This study
**Fig 7**	
*y*^*1*^ w* *P{neoFRT}19A*	[44]
*y*^*1*^ w^*^ *ben*^*A*^ *P{neoFRT}19A*	FBst0057057 [44]
*y*^*1*^ *lrpprc2* ^*A*^ w* *P{neoFRT}19A*	FBst0067166 [39],[44]
*y*^*1*^ *lrpprc2* ^*A*^ *ben*^*A*^ *P{neoFRT}19A*	This study
*Ubiquitin>GFP hsFLP [122]*, *P{neoFRT}19A*	Hugo Bellen
**Fig 8**	
*w;;P{GawB}C57*, *P{UAS-DsRed*.*mito}*	Hugo Bellen
*W;; P{UASp-mito-PA-GFP}*	[73]
*y*^*1*^ w^*^ *ben*^*A*^ *P{neoFRT}19A**;;P{GawB}C57*, *P{UAS-DsRed*.*mito}*	This study
*y*^*1*^ *lrpprc2* ^*A*^ w* *P{neoFRT}19A**;;P{GawB}C57*, *P{UAS-DsRed*.*mito}*	This study
*y*^*1*^ *lrpprc2* ^*A*^ *ben*^*A*^ *P{neoFRT}19A**;;P{GawB}C57*, *P{UAS-DsRed*.*mito}*	This study
**Fig 9**	
*y*^*1*^ w* *P{neoFRT}19A*	[44]
*y*^*1*^ w^*^ *ben*^*A*^ *P{neoFRT}19A*	FBst0057057 [44]
*y*^*1*^ *lrpprc2* ^*A*^ w* *P{neoFRT}19A*	FBst0067166 [39]
*y*^*1*^ *lrpprc2* ^*A*^ *ben*^*A*^ *P{neoFRT}19A*	This study
*cl*^*1*^ w^*^ *FRT19A/ Dp(1;Y)y+ v+; ey-FLP*	[44]
**S1 Fig**	
*y*^*1*^ *lrpprc2* ^*A*^ w* *P{neoFRT}19A*	FBst0067166 [39]
*y*^*1*^ *lrpprc2* ^*E*^ w* *P{neoFRT}19A*	FBst0067167 [39]
*P{Ubi-mRFP*.*nls}*, w* P{hsFLP}, *P{neoFRT}19A*	FBst0031418
w*;*P{w[+mC] = Marf-gHA}2* (Genomic construct Chromosome II)	FBst0067156 [46]
*P{Ubi-mRFP*.*nls}*, w* P{hsFLP}, *P{neoFRT}19A*; *P{Marf-gHA}2*	This study
*Marf*::*mCherry* (Genomic rescue construct, Chromosome III)	Hugo Bellen
*Ubiquitin>GFP*, *hsFLP [122]*, *P{neoFRT}19A**;; Marf*::*mCherry* (III)	This study
*y*^*1*^ w* P{neoFRT}19A	[44]
*Ubiquitin>GFP hsFLP [122]*, *P{neoFRT}19A*	Hugo Bellen
**S2 Fig**	
*Marf*::*mCherry* (Genomic rescue construct, Chromosome III)	Hugo Bellen
*y*^*1*^ *lrpprc2* ^*A*^ w* *P{neoFRT}19A*	FBst0067166 [39],[44]
*Ubiquitin>GFP hsFLP [122]*, *P{neoFRT}19A*	Hugo Bellen
**S3 Fig**	
*y*^*1*^ w* HUWE1^*B*^ *P{neoFRT}19A*	FBst0052343 [44]
lrpprc2 ^*A*^ *HUWE1*^*B*^ *P{neoFRT}19A*	This study
*Ubiquitin>GFP*, *hsFLP [122]*, *P{neoFRT}19A**;; Marf*::*HA* (III)	This study
*MUL1*^*A6*^ (III)	FBal0301081 [57]
*y*^*1*^ *lrpprc2* ^*A*^ w* *P{neoFRT}19A*;; *MUL1*^*A6*^	This study
*Ubiquitin >GFP*, *hsFLP [122]*, *P{neoFRT}19A**;; MUL1*^*A6*^	This study
*P*{*Pink1-9Myc*} (Pink1::Myc) (II)	FBtp0022940 [58]
*Ubiquitin >GFP*, *hsFLP [122]*, *P{neoFRT}19A**; P*{*Pink1-9Myc*} (Pink1::Myc) (II)	This study
**S4 Fig**	
*y*^*1*^ *lrpprc2* ^*A*^ w* *P{neoFRT}19A*	FBst0067166 [39],[44]
*Ubiquitin>GFP hsFLP [122]*, *P{neoFRT}19A*	Hugo Bellen
*w*^*1118*^;*P{w[+mW*.*hs] = en2*.*4-GAL4}e16E*, *P{w[+mC] = UAS-RFP*.*W}2*	FBst0030577
*w*; *P{Marf*::*HA}2* *P{w[+mW*.*hs] = en2*.*4-GAL4}e16E*, *P{w[+mC] = UAS-RFP*.*W}2*	This study
*y*^*1*^ *lrpprc2* ^*A*^ w* *P{neoFRT}19A*;*P{Marf*::*HA}2* *P{w[+mW*.*hs] = en2*.*4-GAL4}e16E*, *P{w[+mC] = UAS-RFP*.*W}2*	This study
*y*^*1*^ *v*^*1*^; *P{y[+t7*.*7] v[+t1*.*8] = TRiP*.*JF02007}attP2* (RNAi against *crc*)	FBst0025985
*Ubiquitin >GFP*, *hsFLP [122]*, *P{neoFRT}19A**;; P{y[+t7*.*7] v[+t1*.*8] = TRiP*.*JF02007}attP2* (RNAi against *crc*)	This study
*w*^*1118*^; *P{GD1425}v3781* (RNAi against *dve*)	FBti0084290
*Ubiquitin >GFP*, *hsFLP [122]*, *P{neoFRT}19A**;;* *P{GD1425}v3781* (RNAi against *dve*)	This study
*y*^*1*^ *v*^*1*^; *P{y[+t7*.*7] v[+t1*.*8] = TRiP*.*JF02734}attP2* (RNAi against *foxo*)	FBst0027656
*Ubiquitin >GFP*, *hsFLP [122]*, *P{neoFRT}19A**;; P{y[+t7*.*7] v[+t1*.*8] = TRiP*.*JF02734}attP2* (RNAi against *foxo*)	This study
*y*^*1*^ w*; *P{en2*.*4-GAL4}e16E*	FBst0030564
w[*]; P{w[+mC] = UAS-2xEGFP}AH2	FBti0026662
*P{en2*.*4-GAL4}e16E*, *P{w[+mC] = UAS-2xEGFP}AH2*	Hugo Bellen
*P{UAS-rOTC*.*P} (UAS-OTC)*	FBal0291051 [67]
*P{UAS-rOTC*.*d} (UAS-ΔOTC)*	FBal0291052 [67]
**S5 Fig**	
*P{FRT(w* ^*hs*^ *)}2A*	FBst0001997
*park*^*Δ21*^ *P{FRT(w*^*hs*^*)}2A*	This study
*P{hsFLP}1*, y1 w* P{UAS-mCD8::*GFP*.*L}Ptp4ELL4; P{tubP-GAL80}LL9 P{FRT(whs)}2A*	FBst0044404
*Pink1*^*5*^ *P{neoFRT}19A*	This study
*Ubiquitin>GFP*, *hsFLP [122]*, *P{neoFRT}19A**;; Marf*::*HA* (III)	This study
*y*^*1*^ w^*^ *ben*^*B*^ *P{neoFRT}19A*	FBst0057058 [44]
*P{Ubi-mRFP*.*nls}*, w^*^ *P{hsFLP}*, *P{neoFRT}19A*; P{*Marf*::*HA*}2	This study
*y*^*1*^ *lrpprc2* ^*A*^ w^*^ben^*B*^ *P{neoFRT}19A*	This study
*y*^*1*^ w^*^;;P{w[+mC] = tubP-GAL4}LL7 (III)	FBti0012687
w^*^;;P{UAS.*ben*::*V5*.*w+mC}*	This study
**S6 Fig**	
w^*^; *P{UAS-HA*::*ben*.*w+mC}*	This study
*Marf*::*mCherry* (Genomic rescue construct, Chromosome III)	Hugo Bellen
*y1* w*; *P{en2*.*4-GAL4}e16E*	FBst0030564
w[*]; P{w[+mC] = UAS-2xEGFP}AH2	FBti0026662
*P{en2*.*4-GAL4}e16E*, *P{w[+mC] = UAS-2xEGFP}AH2*	Hugo Bellen
**S7 Fig**	
w^*^; *P{w[+mC] = UAS-Pink1*.*C}A*	FBst0051648 [58]
*w*; *P{Marf*::*HA}2* *P{w[+mW*.*hs] = en2*.*4-GAL4}e16E*, *P{w[+mC] = UAS-RFP*.*W}2*	This study
*y*^*1*^ w^*^ *ben*^*A*^ *P{neoFRT}19A*; *P{Marf*::*HA}2* *P{w[+mW*.*hs] = en2*.*4-GAL4}e16E*, *P{w[+mC] = UAS-RFP*.*W}2*	This study
w^*^; P{sqh-EYFP-Mito}(III)	FBst0007194
*P{Ubi-mRFP*.*nls}*, w^*^ *P{hsFLP}*, *P{neoFRT}19A;; P{sqh-EYFP-Mito}*(III)	This study
*y*^*1*^ *lrpprc2* ^*A*^ w* *P{neoFRT}19A*	FBst0067166 [39],[44]
*Ubiquitin>GFP hsFLP [122]*, *P{neoFRT}19A*	Hugo Bellen
*w*^*1118*^;*P{w[+mW*.*hs] = en2*.*4-GAL4}e16E*, *P{w[+mC] = UAS-RFP*.*W}2*	FBst0030577
*y*^*1*^ *lrpprc2* ^*A*^ w* *P{neoFRT}19A*;*P{Marf*::*HA}2* *P{w[+mW*.*hs] = en2*.*4-GAL4}e16E*, *P{w[+mC] = UAS-RFP*.*W}2*	This study
*Ubiquitin>GFP*, *hsFLP [122]*,*P{neoFRT}19A**;;P{w[+mC] = UAS-Pink1*.*C}A*	This study
*w;;P{UAS-HA-park*.*Y}*	FBal0301084 [57]
**S8 Fig**	
*y*^*1*^ w^*^ *P{neoFRT}19A*	[44]
*y*^*1*^ w^*^ *ben*^*A*^ *P{neoFRT}19A*	FBst0057057
*y*^*1*^ *lrpprc2* ^*A*^ w^*^ *P{neoFRT}19A*	FBst0067166 [39],[44]
*y*^*1*^ *lrpprc2* ^*A*^ w^*^ben^*A*^ *P{neoFRT}19A*	This study
**S9 Fig**	
y^1^ w* P{neoFRT}19A	[44]
y^1^ w* ben^A^ *P{neoFRT}19A*	FBst0057057 [44]
y^1^ lrpprc2 ^A^ w* *P{neoFRT}19A*	FBst0067166 [39]
*y^1^ lrpprc2 ^A^ ben^A^* *P{neoFRT}19A*	This study
*Ubiquitin>GFP hsFLP [122]*, *P{neoFRT}19A**; Ubx-FLP*	Hugo Bellen

For drug treatments, 3^rd^ instar larvae were transferred to food containing 3mg/ml chloroquine [106], 100μM MG132, or DMSO (vehicle control) for 24 hours prior to dissection. For western blot and qPCR, 3^rd^ instar larvae were used. We observed that development of *lrpprc2*^*A*^ mutant larvae is substantially delayed. Therefore, we used size matched 3^rd^ instar *lrpprc2*^*A*^ mutant larvae that are obtained after 14–15 days post hatching.

### Generation of transgenic flies

*ben* sequence was amplified from genomic DNA. These PCR amplified *ben* ORF sequences were then inserted into a pUAST vector containing attB sites, flanking the insert using EcoRI-XhoI. pUAST vectors containing *UAS-ben*::*V5/UAS-HA*::*ben* were injected into embryos containing attP2 landing site and integrase. Transgenic flies were selected based on the presence of w^+mC^. Primers used: P{UAS.*ben*::*V5*.w^+mC^}: Fwd-5’-GGAATTCGCCACCATGTCCAGCC TGCCACGTC-3’ and Rev-5’-CCGCTCGAGTTACGTAGAATCGAGACCGAGGAGA GGGTTAGGGATAGGCTTACCGTCTTCGACGGCATAT-3’. P{UAS-*HA*::*ben*.w^+mC^}:

Fwd-5’-GGAATTCGCCACCATGTACCCATACGACGTCCCAGACTACGCTATGTCCAGCCTGCCACGTC-3’ and Rev-5’-CCGCTCGAGTCAGTCTTCGACGGCATAT-3’.

Opa1::3FLAG-2HA genomic construct was generated using the P(acman) system [107]. Briefly, the 3FLAG-2HA tag was amplified from C-terminal tag fusion vector pL452-C-3FLAG-2HA and inserted at the C terminal of Opa1 through recombineering in the P(acman) clone CH322-27B08, which was subsequently injected into *y*^*1*^ *w*^*1118*^; PBac{y+-attP-3B}VK00033 flies.

### Immunofluorescence and imaging

Larvae were dissected in 1X PBS, followed by fixing in 4% paraformaldehyde (Himedia—TCL-119 - 100ml) for 30 minutes at room temperature and three washes in 1X PBS with 0.2% TritonX-100 (Himedia—MB031, 1X PBST). Primary antibodies were incubated overnight at 4°C. Followed by blocking in 5% normal goat serum (Himedia—RM10701) for 1h at room temperature and then secondary antibody incubation followed by washing and dissection. Samples were mounted in Vectashield (VectorLabs—H100) and imaged under 40X or 63X oil immersion Leica Stellaris 5 or Olympus FV3000 confocal microscopes. Images were processed using Fiji. All antibody dilutions and the blocking solution were made in 1X PBST; details of antibodies and their dilutions used are listed in Table 2.

**Table 2:** Antibodies used in this study.

Antibody	Catalog	Dilution
Mouse HA	Cell Signaling Technology- 2367S	1:500 IF
Rabbit HA	Cell Signaling Technology- 3724S	1:500 IF
Mouse Complex V	Abcam- 176569	1:500 IF 1:2500 WB
Rabbit V5	Cell Signaling Technology- 13202S	1:500 IF 1: 5000 WB
Rabbit mCherry	Cell Signaling Technology- 43590S	1:500 IF
Rabbit Actin	Cell Signaling Technology- 4967S	1:5000 WB
Mouse Actin	Invitrogen- MA5- 15739	1:5000 WB
Rabbit Tubulin	Novus Biologicals- NB100-56459	1:1000 WB
Rabbit Hsp60A	[112]	1:200 IF
Rabbit Hsp60	Cell Signaling Technology- 4870S	1:500 IF
Mouse Myc Tag	Cell Signaling Technology-2276S	1:1000 WB 1:500 IF
Mouse Myc Tag	NB600-302	1:1000 WB
Rabbit p62	Abcam- ab178440	1:500 IF
Anti-Mouse 488	Invitrogen- A11029	1:500 IF
Anti Mouse 555	Invitrogen- A21424	1:500 IF
Anti-Mouse 633	Invitrogen- A21052	1:500 IF
Anti-Rabbit 488	Invitrogen- A32731	1:500 IF
Anti-Rabbit 555	Invitrogen- A32732	1:500 IF
Anti-Rabbit 647	Invitrogen- A32733	1:500 IF
Anti-Rabbit HRP	Novus Biologicals- NB7160	1:5000 WB
Anti-Mouse HRP	Novus Biologicals- NB7539	1:5000 WB

IF- Immunofluorescence WB- Western Blot

### Eye and wing phenotype imaging

Mutant eyes were created by crossing heterozygous mutant flies with *w cl(1)* FRT19A /Dp(1;Y); *ey-FLP* flies. The eye images were then acquired on a Leica M205FA Stereo Zoom microscope.

Wing clones were made by crossing heterozygous mutant flies with using *UbiGFP frt19A*; *Ubx-FLP*. The flies were anesthetised and fixed in 70% ethanol and stored at 4°C. For mounting, the wings were dissected from the flies and transferred to 100% ethanol. The wings were then mounted in DPX (Sigma 06522-100mL). The DPX media was allowed to evenly spread by applying weight on the coverslip and incubating the slides at 60°C. The samples were imaged using transmitted light in a Leica- DMi8 inverted microscope using a 4X objective.

### Western blot

3^rd^ instar larvae were crushed in RIPA lysis buffer [50mM Tris,150mM NaCl, 0.2% Triton X 100 and 1X protease and phosphatase inhibitor cocktail (Thermo Fisher—A32965, A32957 respectively)], followed by centrifugation at 16,000g for 10 mins at 4°C. Clear fat-free supernatant was used for total protein estimation by BCA assay (Thermo Scientific—23227). Lysate was mixed with equal volume of 1X Laemmli buffer (0.004% bromophenol blue, 20% glycerol, 4% SDS and 0.125M Tris-HCl pH 6.8) having 5% beta-mercaptoethanol and heated at 98°C for 5 minutes, centrifuged, and 25μg of protein was loaded in each well and resolved on 4–15% gradient Tris-Glycine gel (Bio-Rad—4561086). Semi-dry transfer was done onto 0.2μm Nitrocellulose membrane as per Trans-BlotTurbo Kit (Bio-Rad—1704270) for seven minutes. Blocking in either 5% Blotto (Santa Cruz sc—2325) or 5% BSA made in 1X TBS with 0.1% Tween-20 (1X TBSTw20) for 1 hour at room temperature followed by primary antibody incubation overnight at 4°C. After washing thrice in 1X TBSTw20, membranes were incubated in HRP conjugated secondary antibodies (Table 2) for 2 hours at room temperature. After washing, they were developed using Clarity Western ECL Substrate (Bio-Rad—1705061) and visualized using Vilber-Lourmat chemidoc. Band intensities were quantified using Fiji and normalized with Actin.

### Co-immunoprecipitation

25–30 adult flies were homogenized thoroughly in 200ul of co-immunoprecipitation (co-IP) buffer (40mM HEPES pH 7.5, 120mM NaCl, 1mM EDTA, 10mM pyrophosphate, 10mM glycerophosphate, 50mM NaF, 1mM orthovanadate and 0.3% CHAPS) and incubated for 30 mins on ice. Then centrifuged at 16,000g for 10 mins at 4°C and the supernatant was aspirated out carefully. Protein estimation was done by using the BCA method (Thermo Scientific—23227). To the remaining beads 300ul co-IP buffer was added and incubated with equilibrated 35μl Myc beads (Sigma—E6654) for 4 hrs at 4°C. It was then centrifuged and the supernatant discarded. Beads were washed thrice with the co-IP buffer. The beads were then incubated with 50μl anti-Myc peptide and incubated for 4 hrs at 4°C. After that the sample was centrifuged and supernatant having elute was collected carefully in fresh tube and processed for Western blot as given above.

### Real-time PCR

3^rd^ instar larvae were used for RNA isolation using TRIzol (Ambion life tech—15596018) method. cDNA conversion for 1μg of RNA was carried out using a cDNA conversion kit (Thermo Fisher—4368814). qPCR was carried out in 96 well plates in three technical replicates for each of the three biological replicates. *Marf* qPCR was done using the iTaq SYBR Green supermix (Bio-Rad -1725121) using LightCycler 96 (Fig 5F).

Following primers were used:

Marf-Fwd-5’-CGAGTGCCAGGAATCGGTTA-3’, Marf-Rev5’-ATCTGAAAGCCCTCGGCAAT-3’, RP49-Fwd-5’-TCCTACCAGCTTCAAGATGAC-3’,

RP49-Rev-5’-CACGTTGTGCACCAGGAACT-3’.

### TMRE and mitotracker red staining

3^rd^ instar larvae were dissected in Schneider’s Insect media (Himedia—IML003-500ml). The larvae were transferred to media containing 100nM TMRE (Thermo Fisher—T669) or 200nM MitoTracker Red FM (Invitrogen-M22425) in Schneider’s insect media and incubated for 20 mins. The wing discs were dissected and mounted in Schneider’s media using a coverslip. The tissues were live imaged using Leica Stellaris 5 confocal microscope at 63X oil objective. For mitochondrial morphology analysis, the number of ring shaped and aggregated mitochondria in each cell were counted manually using the Fiji-cell counter Plugin. The numbers per cell were then plotted. The averages were compared using one-way ANOVA-Bonferroni’s Multiple Comparison Test.

### Mitochondrial morphology analysis

Wing discs immunostained for Complex V were imaged using Leica Stellaris 5 confocal microscope at 63X oil objective. The mitochondria were segmented on Fiji using the Trainable Weka segmentation plugin [108]. The segmented images were then used to find out the mitochondrial area using Particle Analyze Tool on Fiji.

Blind test: for qualitative assessment of mitochondrial morphology in larval muscle, we renamed a set of images containing mitochondria from larval muscles with random numbers. The images from different genotypes (*control*, *ben*^*A*^, *lrpprc2*^*A*^, and *lrpprc2*^*A*^ *ben*^*A*^) were pooled and were assessed for the presence of different mitochondrial morphologies, including presence or absence of mitochondria network, large globular mitochondria, ring-shaped mitochondria and mitochondrial aggregates. Multiple images were used for the assessment, 40 images from 11 larvae for control, 24 images from 7 larvae for *ben*^*A*^, 27 images from 7 larvae for *lrpprc2*^*A*^, and 32 images from 9 larvae for *lrpprc2*^*A*^*ben*^*A*^.

### Mitochondrial morphology analysis using mito-PA-GFP

Third instar larvae expressing *C57>Gal4*, *UAS-mito-dsRed*, and *UAS-mito-PA-GFP* were fileted in Schneider’s *Drosophila* Medium. Before imaging, fresh Schneider’s medium with 5mM glutamate was added to block neurally evoked muscle contractions. Imaging was done using Leica Stellaris 5 confocal using a 63X water dipping objective. To activate GFP, a 405nm laser at 100% power was used at designated ROIs for 10 iterations. After activation, 5 images were acquired at 3min intervals in RFP and GFP channels. The images were segmented using the Fiji-Trainable Weka segmentation plugin. The segmented images were used to get various mitochondrial morphology parameters using the Fiji-Mitochondria Analyzer plugin. Parameters including branch number (proxy for network), aspect ratio- ratio of the major axis to the minor axis of a mitochondria (proxy for globular *v/s* tubular) and Area (proxy for size) were then compared using one-way ANOVA-Bonferroni’s Multiple Comparison Test. The photoactivation protocol was modified from Chowdhary et. al. 2017 [73].

### Statistics analysis

At least three independent experiments were used for all quantifications, the n values for each experiment is indicated in their respective figure legends. n represents the number of clones/regions used for the analysis. Two-tailed unpaired t-test was used to analyze data obtained from clonal analysis; One sample t-test was used to analyze the data in S5 Fig. Two-tailed unpaired t-test was used to analyze all other data sets. ANOVA-Bonferroni’s Multiple Comparison Test was used to compare data sets in Figs 5C”, 7I, 7J, 8E, 8F and 8G. Significance of the data was represented as * for p<0.05, ** for p<0.01, and *** for p<0.0001. All statistical analyses were carried out using GraphPad Prism software version 9.

## Supporting information

S1 Fig (*A)* Schematic to illustrate *FLP*-FRT mediated recombination system. *(A’)* Green marks wildtype and heterozygous cells (solid white line, +/+ and +/-), absence of GFP (non green) marks mutant clones/cells (dashed white line, -/-). Created using Biorender.com. *(B-B’) lrpprc2*^*E*^ mutant clones (non green cells, *B* and dashed white line, *B’*), wing discs immunostained for Marf::HA (red). (*C-C’) lrpprc2*^*A*^ mutant clones (non green cells, *C* and dashed white line, *C’*), wing discs immunostained for Marf::mCherry (red). Scale bar represents 20μm. *(B”* and *C”)* Quantification for relative fluorescence intensities of Marf::HA (*B”*, n = 16) and Marf::mCherry (*C”*, n = 5). Graphs represent average intensity values normalized to that of control cells. Two-tailed unpaired t-test between control and *lrpprc2* mutant cells. *(D-D”) lrpprc2*^*A*^ mutant clones (non green cells, *D*) in peripodial cells of third instar larval wing discs, immunostained for Complex V (gray). Inset of control *(D’)* and *lrpprc2*^*A*^ mutant cell *(D”)* from *(D)*. *(D’* and *D”)* Binary image of Complex V staining in control *(D’)* and *lrpprc2*^*A*^ mutant cell *(D”)*. Scale bar represents 10μm in *(D)* and 4μm in *(D’ and D”)*. *(E)* Quantification for area of individual mitochondria in *lrpprc2*^*A*^ mutant clones compared to control cells (n = 6). Two-tailed unpaired t-test was done. Error bars represent S.E.M. Significance represented by p<0.05*, p<0.01*, p<0.001***.
(TIF)

S2 Fig *(A-B’)* Wing discs expressing endogenous Marf::mCherry in control*(A)* and larvae treated with chloroquine*(A’)*, DMSO*(B)* or MG132*(B’)*. *(A”* and *B”)* Quantification for relative fluorescence intensities of Marf::mCherry in control (*A”*, n = 12) and chloroquine treated larvae (*A”*,n = 10) and DMSO (*B”*,n = 12) and MG132 (*B”*,n = 12) treated larvae. Graphs represent average intensity values normalized to control/DMSO. Two tailed unpaired t-test between control and chloroquine and between DMSO and MG132 treatments. *(C-C’) lrpprc2*^*A*^ mutant clones (non green cells, *C* and dashed white line, *C’*), wing discs immunostained for endogenous p62 (red). Scale bar represents 20μm. *(C”)* Quantification for relative fluorescence intensities of p62 in *lrpprc2*^*A*^ mutant clones (n = 12). Graphs represent average intensity values normalized to that of control cells. Two tailed unpaired t-test between control and *lrpprc2*^*A*^ mutant cells. Significance represented by n.s.—non significant, p<0.05 *.
(TIF)

S3 Fig *(A-A’) lrpprc2*^*A*^ *HUWE1*^*B*^ double mutant clones (non green cells, *A* and dashed white line, *A’*), wing discs immunostained for Marf::HA (red). *(B-B’) lrpprc2*^*A*^ mutant clones (non green cells, *B* and dashed white line, *B’*) in *MUL1*^*A6*^ mutant background, wing discs immunostained for Marf::HA (red). *(C-C’) lrpprc2*^*A*^ mutant clones (non green cells, *C* and dashed white line, *C’*), wing discs immunostained for PINK1::Myc (red). *(D-D’) lrpprc2*^*A*^ mutant clones (non green cells, *D* and dashed white line, *D’*), wing discs stained for TMRE (red) and live imaged. Scale bar represents 20μm. *(A”*, *B”*, *C”* and *D”)* Quantification for relative fluorescence intensities of Marf::HA in *lrpprc2*^*A*^ *HUWE1*^*B*^ double mutant clones (*A”*, n = 13), *lrpprc2*^*A*^ mutant clones in *MUL1*^*A6*^ mutant background (*B”*, n = 20), PINK1::Myc in *lrpprc2*^*A*^ mutant clones (*C”*, n = 12) and TMRE in *lrpprc2*^*A*^ mutant clones (*D”*, n = 20). Graphs represent average intensity values normalized to that of control/*MUL1*^*A6*^ cells. Two-tailed unpaired t-test between control/*MUL1*^*A6*^ and mutant cells. Significance represented by n.s.- non significant, p<0.001***.
(TIF)

S4 Fig *(A-A’) lrpprc2*^*A*^ mutant clones (non green cells, *A* and dashed white line, *A’*), wing discs immunostained for Hsp60 (red). *(B-D’) lrpprc2*^*A*^ mutant clones (non green cells, B,*C*, *D* and dashed white line, *B’ C’*, *D’*) on knockdown of *crc(B-B’)*, *foxo(C-C’)* and *dve(D-D’)* using *En>Gal4*, wing discs marked by *UAS-RFP* (green) and immunostained for Marf::HA (red). *(E-E’)* Overexpression of *ΔOTC* using *En>Gal4*, wing discs marked by *UAS-RFP* (green) and immunostained for Hsp60 (red). *(F-G”)* Overexpression of *OTC(F-F’*) and *ΔOTC(G-G’)* using *En>Gal4*, wing discs marked by *UAS-RFP* (green) and immunostained for Marf::HA (red). Scale bar represents 20μm. *(A”-G”)* Quantification for relative fluorescence intensities of Hsp60 in *lrpprc2*^*A*^ mutant clones (*A”*, n = 14), Marf::HA in *lrpprc2*^*A*^ mutant clones on knockdown of *crc*(*B”*, n = 6), *foxo*(*C”*,n = 8) and *dve*(*D”*,n = 10), Hsp60 on *UAS-ΔOTC* expression (*E”*, n = 6) and Marf::HA on *UAS-OTC* expression (*F”*, n = 9) and *UAS-ΔOTC* expression (*G”*,n = 18). Graphs represent average intensity values normalized to that of control cells. Two-tailed unpaired t-test between control and *lrpprc2*^*A*^ mutant cells/ cells overexpressing UAS-OTC or UAS-ΔOTC. Significance represented by n.s.—non significant, p<0.01**, p<0.001***.
(TIF)

S5 Fig *(A-C’)* Wing discs immunostained for Marf::HA (red) in *park*^*Δ21*^ mutant clones (green cells, *A-A’)*, *Pink1*^*5*^ mutant clones, *ben*^*B*^ mutant clones and *lrpprc2*^*A*^ *ben*^*B*^ double mutant clones (non green cells, *B*,*C*, *D* and dashed white line, *B’*,*C’*,*D’*). Scale bar represents 20μm. *(A”*, *B”*, *C”* and *D”)* Quantification for relative fluorescence intensities of Marf::HA in *park*^*Δ21*^ (n = 14), *Pink1*^*5*^ (n = 15), *ben*^*B*^ (n = 14) and *lrpprc2*^*A*^ *ben*^*B*^ double mutant clones (*D”*, n = 15). Graphs represent average intensity values normalized to that of control cells. Two-tailed unpaired t-test between control and mutant cells. Significance represented by p<0.05*, p<0.01**, p<0.0001*** *(E)* Identity and similarity between Ben and its homologs. (*F)* Ben mutations and lethal staging. *(G)* Schematic showing point mutations in *ben*^*A*^ and *ben*^*B*^ alleles.
(TIF)

S6 Fig *(A-A’)* Wing discs immunostained for Marf::mCherry (red) on overexpression of *HA*::*Ben* using *En>Gal4*, wing discs marked with *UAS-GFP* (green). *(A”)* Quantification for relative fluorescence intensities of Marf::mCherry on overexpression of *HA*::*ben* (n = 12). Graphs represent average intensity values normalized to that of control cells. Two-tailed unpaired t-test between control and *UAS-HA*::*Ben* overexpressing cells. n.s—non significant.
(TIF)

S7 Fig *(A-A’) ben*^*A*^ mutant on overexpression of *Pink1* using *En*>*Gal4*, wing discs marked with *UAS-RFP* (blue) and immunostained for Marf::HA (red). *(B-B’) ben*^*A*^ mutant clone (non-green cell, *B* and dashed white line, *B’*), pupal gut 2h APF expressing *Sq>mito-EYFP* (red). *(C-C’)* Overexpression of *Pink1* using *En*>*Gal4*, wing discs marked with *UAS-RFP* (blue) and immunostained for Complex V (red). *(D-D’)* Overexpression of *Park* using *En*>*Gal4*, wing discs immunostained for HA (green) and Complex V (red). Scale bar represents 20μm. (*C”* and *D”*) Quantification for relative fluorescence intensities of Complex V in *UAS-Pink1* cells (*C”*, n = 15) and *UAS-HA*::*Park* cells (*D”*, n = 15). Graphs represent average intensity values normalized to that of control. Two-tailed unpaired t-test between control and cells overexpressing *UAS-Pink1/UAS-HA*::*Park*. *(E-E”)* Overexpression of *Pink1* using *En*>*Gal4*, wing discs marked with *UAS-RFP* (blue, *E*) and immunostained for Marf::HA (red, *E-E”*) with *lrpprc2*^*A*^ mutant clone (non-green cell, *E*, *E’* and *E”* and dashed white line, *E’* and *E”*). *(F)* Average Marf::HA intensity values in wildtype, *lrpprc2*^*A*^ mutant clones, *UAS-Pink1* and *lrpprc2*^*A*^ mutant clones in *UAS-Pink1* background, normalized to that of control cells (non RFP expressing GFP positive cells). A one-way ANOVA-Bonferroni’s multiple comparison test was used to calculate the significance between the samples in graph *F*. Error bars represent S.E.M. Significance represented by n.s.- non significant, p<0.05*, p<0.01**, p<0.0001***.
(TIF)

S8 Fig *(A-D)* Confocal sections of third instar larval muscles immunostained for endogenous Complex V (gray) in control*(A)*, *ben*^*A*^*(B)*, *lrpprc2*^*A*^*(C)* and *lrpprc2*^*A*^ *ben*^*A*^*(D)* larvae.
(TIF)

S9 Fig *(A-D)* Mutant wing clones from young flies of control*(A-A”)*, *ben*^*A*^*(B-B”)*, *lrpprc2*^*A*^*(C-C”)*, and *lrpprc2*^*A*^ *ben*^*A*^*(D-D”)* genotypes. Ectopic veins are marked by yellow arrowheads. Scale bar represents 1mm *(A-D)*, 200μm *(A’-D’)* and 100μm *(A”-D”)* The images within the figure panels are created by the authors.
(TIF)

S1 Data All numerical data underlying the graphs are provided in the S1 Data.xlsx file. (XLSX)
