# Correction: Bendless is essential for PINK1-Park mediated Mitofusin degradation under mitochondrial stress caused by loss of LRPPRC

**DOI:** 10.1371/journal.pgen.1012268

**Published:** 2026-08-05

**Authors:** Rajit Narayanan Cheramangalam, Tarana Anand, Priyanka Pandey, Deepa Balasubramanian, Reshmi Varghese, Neha Singhal, Sonal Nagarkar Jaiswal, Manish Jaiswal

An additional affiliation is missing for the third and seventh author. Priyanka Pandey and Sonal Nagarkar Jaiswal are also affiliated with the Academy of Scientific and Innovative Research, Ghaziabad 201002, India.
