# Supplementary figures and images for: Bendless is essential for PINK1-Park mediated Mitofusin degradation under mitochondrial stress caused by loss of LRPPRC
Source: PLoS Genet. 2023 Apr 25;19(4):e1010493. doi: 10.1371/journal.pgen.1010493 (PMC10162545; doi:10.1371/journal.pgen.1010493)

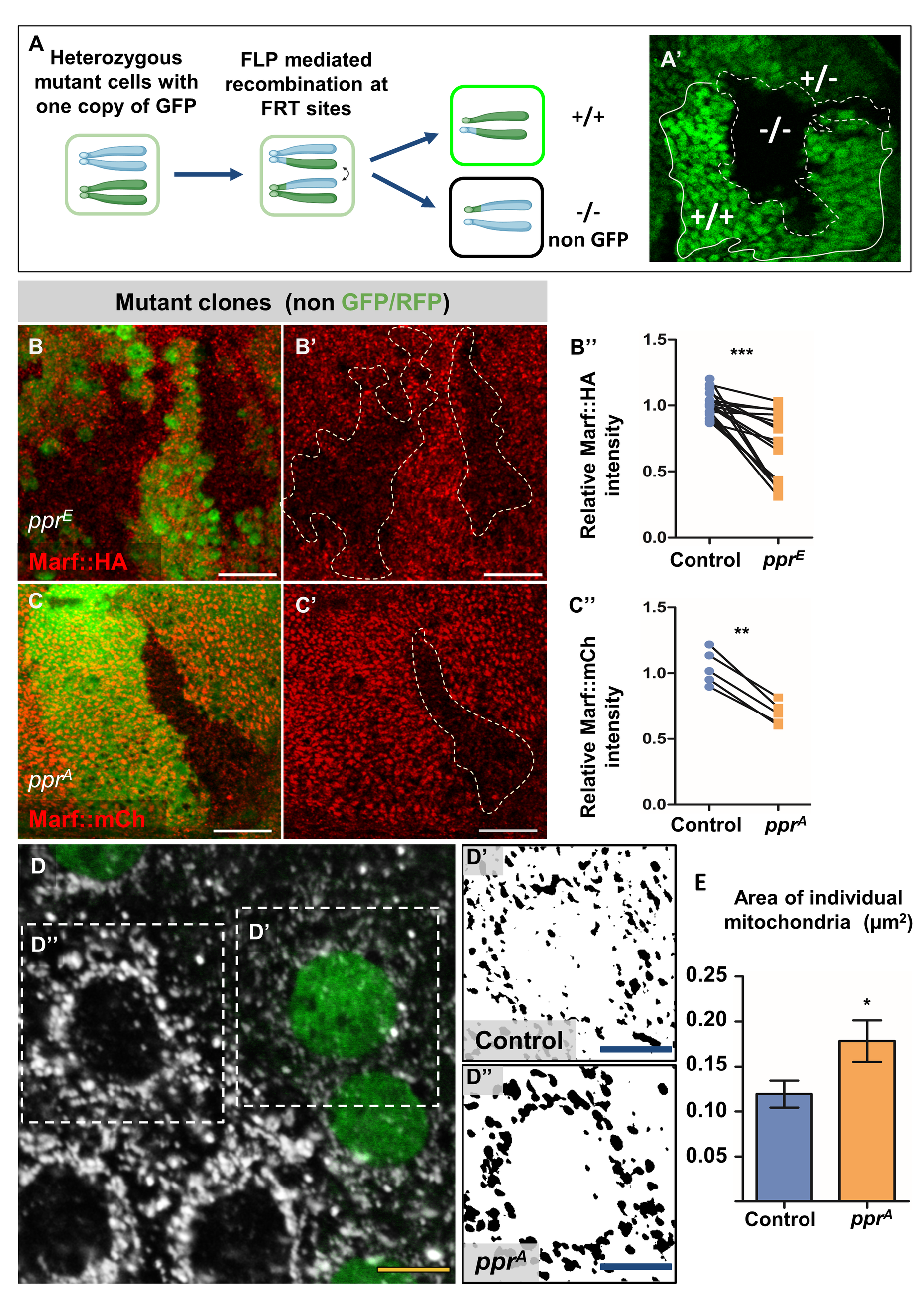

Supplement: S1 Fig — (A) Schematic to illustrate FLP-FRT mediated recombination system. (A’) Green marks wildtype and heterozygous cells (solid white line, +/+ and +/-), absence of GFP (non green) marks mutant clones/cells (dashed white line, -/-). Created using Biorender.com. (B-B’) lrpprc2E mutant clones (non green cells, B and dashed white line, B’), wing discs immunostained for Marf::HA (red). (C-C’) lrpprc2A mutant clones (non green cells, C and dashed white line, C’), wing discs immunostained for Marf::mCherry (red). Scale bar represents 20μm. (B” and C”) Quantification for relative fluorescence intensities of Marf::HA (B”, n = 16) and Marf::mCherry (C”, n = 5). Graphs represent average intensity values normalized to that of control cells. Two-tailed unpaired t-test between control and lrpprc2 mutant cells. (D-D”) lrpprc2A mutant clones (non green cells, D) in peripodial cells of third instar larval wing discs, immunostained for Complex V (gray). Inset of control (D’) and lrpprc2A mutant cell (D”) from (D). (D’ and D”) Binary image of Complex V staining in control (D’) and lrpprc2A mutant cell (D”). Scale bar represents 10μm in (D) and 4μm in (D’ and D”). (E) Quantification for area of individual mitochondria in lrpprc2A mutant clones compared to control cells (n = 6). Two-tailed unpaired t-test was done. Error bars represent S.E.M. Significance represented by p<0.05*, p<0.01*, p<0.001***. (TIF) [file pgen.1010493.s001.tif]

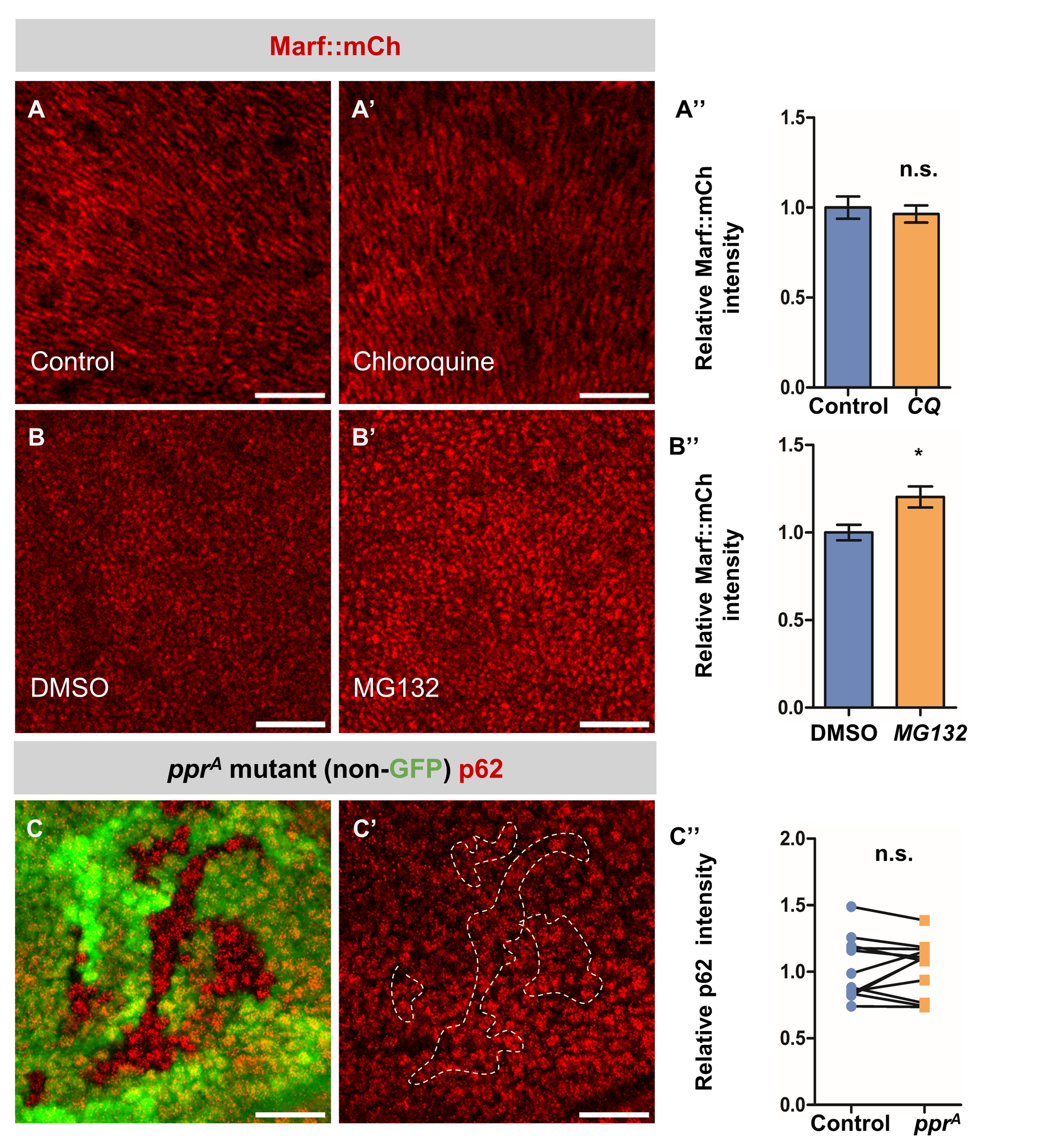

Supplement: S2 Fig — (A-B’) Wing discs expressing endogenous Marf::mCherry in control(A) and larvae treated with chloroquine(A’), DMSO(B) or MG132(B’). (A” and B”) Quantification for relative fluorescence intensities of Marf::mCherry in control (A”, n = 12) and chloroquine treated larvae (A”,n = 10) and DMSO (B”,n = 12) and MG132 (B”,n = 12) treated larvae. Graphs represent average intensity values normalized to control/DMSO. Two tailed unpaired t-test between control and chloroquine and between DMSO and MG132 treatments. (C-C’) lrpprc2A mutant clones (non green cells, C and dashed white line, C’), wing discs immunostained for endogenous p62 (red). Scale bar represents 20μm. (C”) Quantification for relative fluorescence intensities of p62 in lrpprc2A mutant clones (n = 12). Graphs represent average intensity values normalized to that of control cells. Two tailed unpaired t-test between control and lrpprc2A mutant cells. Significance represented by n.s.—non significant, p<0.05 *. (TIF) [file pgen.1010493.s002.tif]

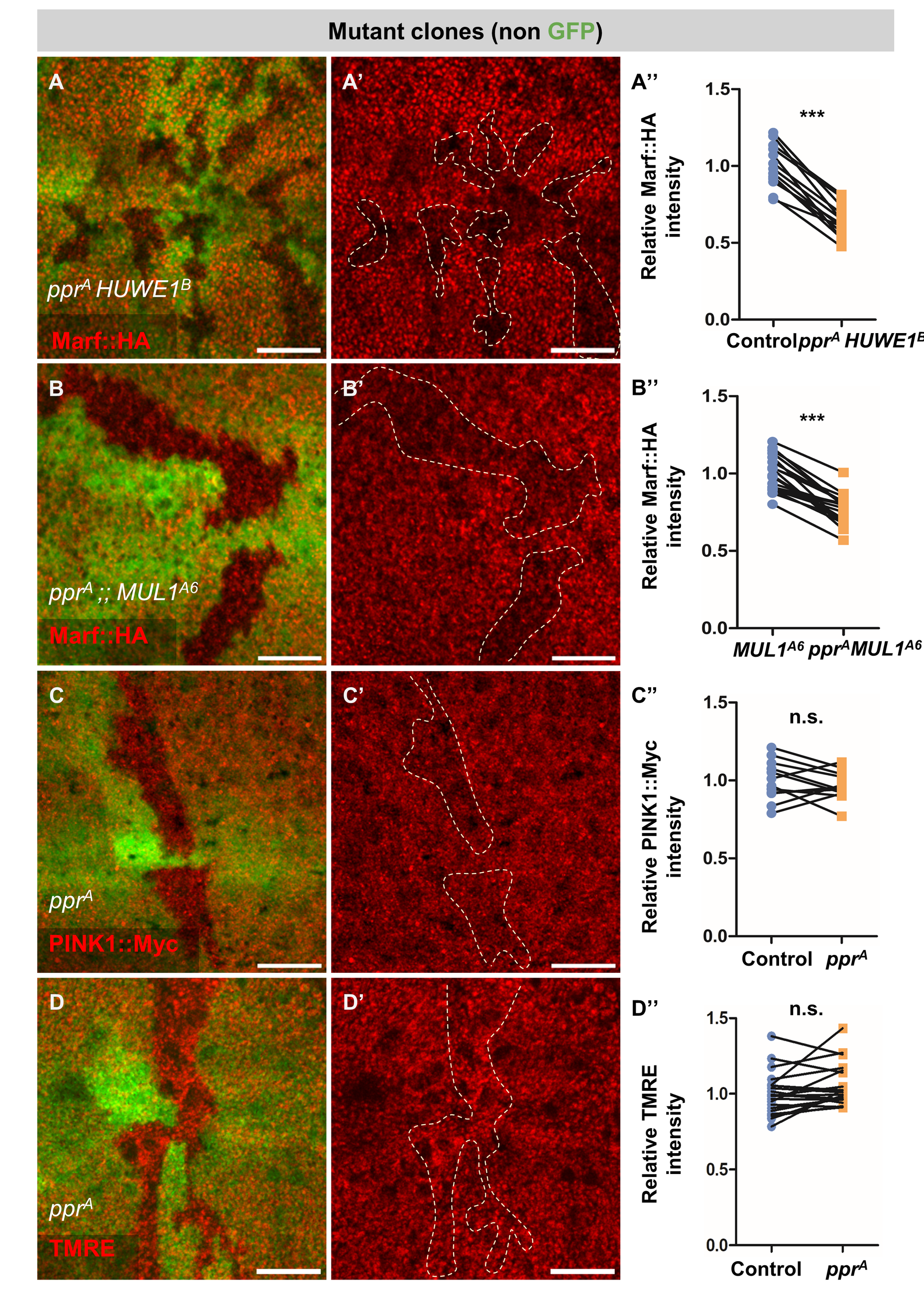

Supplement: S3 Fig — (A-A’) lrpprc2A HUWE1B double mutant clones (non green cells, A and dashed white line, A’), wing discs immunostained for Marf::HA (red). (B-B’) lrpprc2A mutant clones (non green cells, B and dashed white line, B’) in MUL1A6 mutant background, wing discs immunostained for Marf::HA (red). (C-C’) lrpprc2A mutant clones (non green cells, C and dashed white line, C’), wing discs immunostained for PINK1::Myc (red). (D-D’) lrpprc2A mutant clones (non green cells, D and dashed white line, D’), wing discs stained for TMRE (red) and live imaged. Scale bar represents 20μm. (A”, B”, C” and D”) Quantification for relative fluorescence intensities of Marf::HA in lrpprc2A HUWE1B double mutant clones (A”, n = 13), lrpprc2A mutant clones in MUL1A6 mutant background (B”, n = 20), PINK1::Myc in lrpprc2A mutant clones (C”, n = 12) and TMRE in lrpprc2A mutant clones (D”, n = 20). Graphs represent average intensity values normalized to that of control/MUL1A6 cells. Two-tailed unpaired t-test between control/MUL1A6 and mutant cells. Significance represented by n.s.- non significant, p<0.001***. (TIF) [file pgen.1010493.s003.tif]

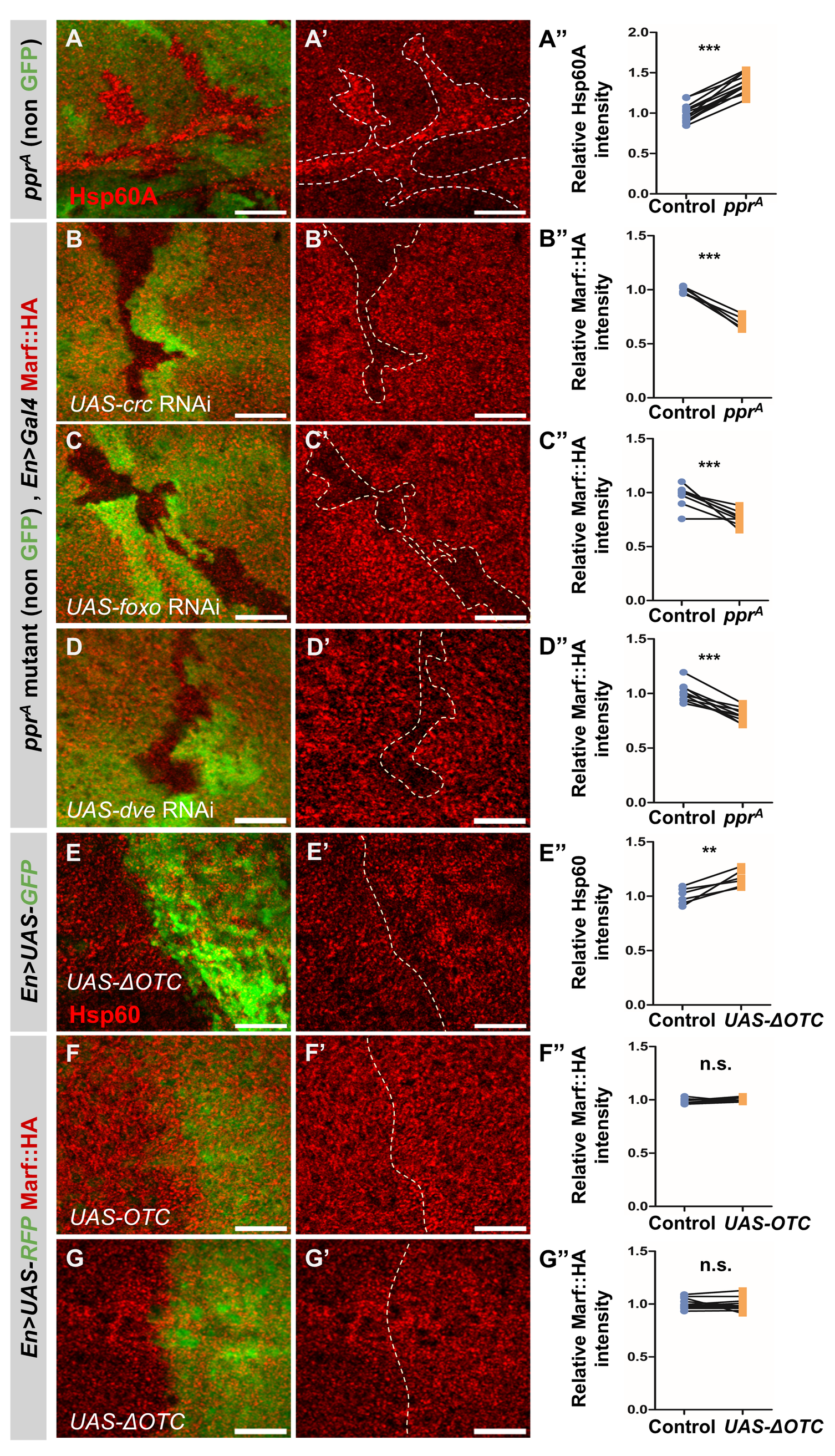

Supplement: S4 Fig — (A-A’) lrpprc2A mutant clones (non green cells, A and dashed white line, A’), wing discs immunostained for Hsp60 (red). (B-D’) lrpprc2A mutant clones (non green cells, B,C, D and dashed white line, B’ C’, D’) on knockdown of crc(B-B’), foxo(C-C’) and dve(D-D’) using En>Gal4, wing discs marked by UAS-RFP (green) and immunostained for Marf::HA (red). (E-E’) Overexpression of ΔOTC using En>Gal4, wing discs marked by UAS-RFP (green) and immunostained for Hsp60 (red). (F-G”) Overexpression of OTC(F-F’) and ΔOTC(G-G’) using En>Gal4, wing discs marked by UAS-RFP (green) and immunostained for Marf::HA (red). Scale bar represents 20μm. (A”-G”) Quantification for relative fluorescence intensities of Hsp60 in lrpprc2A mutant clones (A”, n = 14), Marf::HA in lrpprc2A mutant clones on knockdown of crc(B”, n = 6), foxo(C”,n = 8) and dve(D”,n = 10), Hsp60 on UAS-ΔOTC expression (E”, n = 6) and Marf::HA on UAS-OTC expression (F”, n = 9) and UAS-ΔOTC expression (G”,n = 18). Graphs represent average intensity values normalized to that of control cells. Two-tailed unpaired t-test between control and lrpprc2A mutant cells/ cells overexpressing UAS-OTC or UAS-ΔOTC. Significance represented by n.s.—non significant, p<0.01**, p<0.001***. (TIF) [file pgen.1010493.s004.tif]

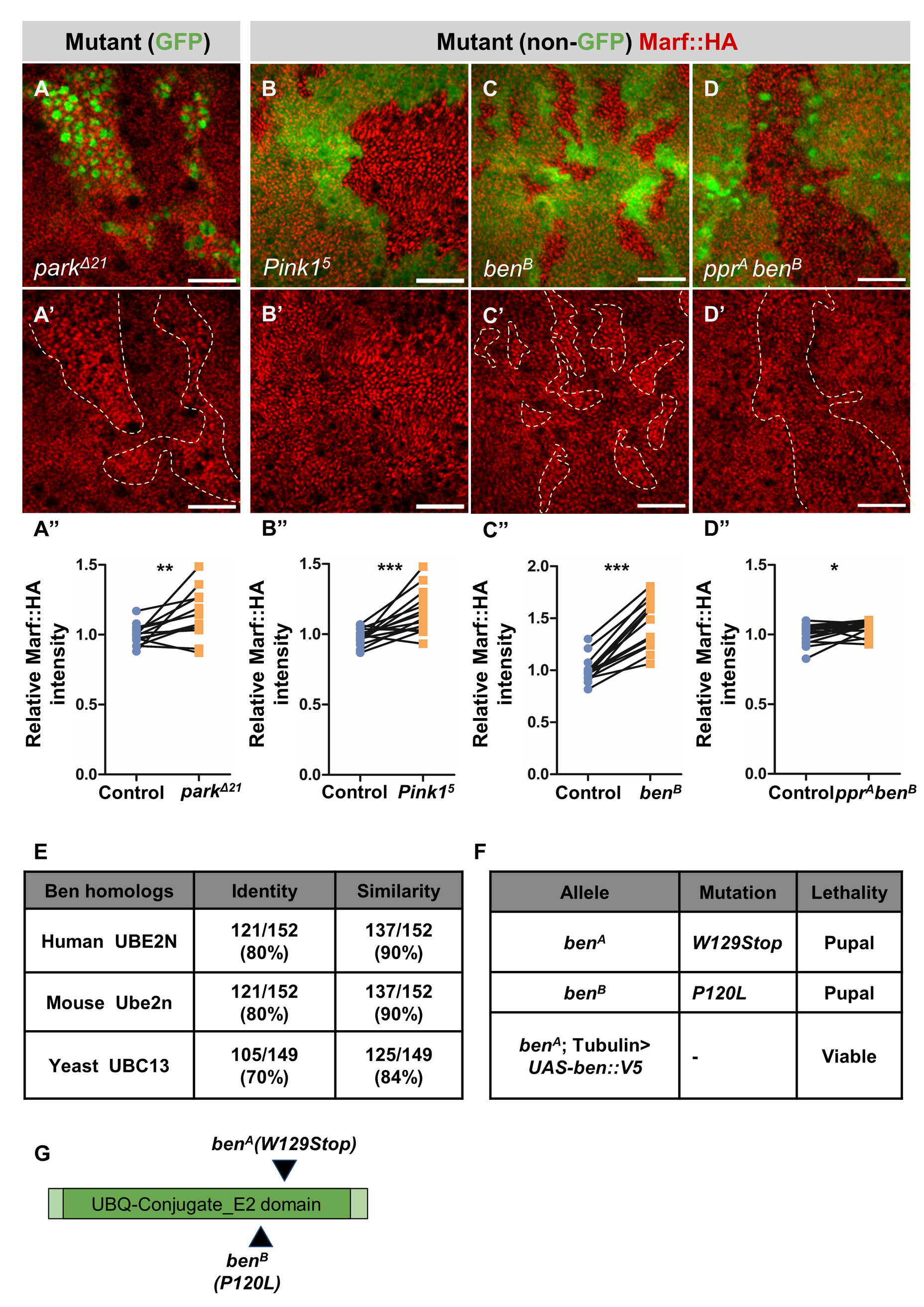

Supplement: S5 Fig — (A-C’) Wing discs immunostained for Marf::HA (red) in parkΔ21 mutant clones (green cells, A-A’), Pink15 mutant clones, benB mutant clones and lrpprc2A benB double mutant clones (non green cells, B,C, D and dashed white line, B’,C’,D’). Scale bar represents 20μm. (A”, B”, C” and D”) Quantification for relative fluorescence intensities of Marf::HA in parkΔ21 (n = 14), Pink15 (n = 15), benB (n = 14) and lrpprc2A benB double mutant clones (D”, n = 15). Graphs represent average intensity values normalized to that of control cells. Two-tailed unpaired t-test between control and mutant cells. Significance represented by p<0.05*, p<0.01**, p<0.0001*** (E) Identity and similarity between Ben and its homologs. (F) Ben mutations and lethal staging. (G) Schematic showing point mutations in benA and benB alleles. (TIF) [file pgen.1010493.s005.tif]

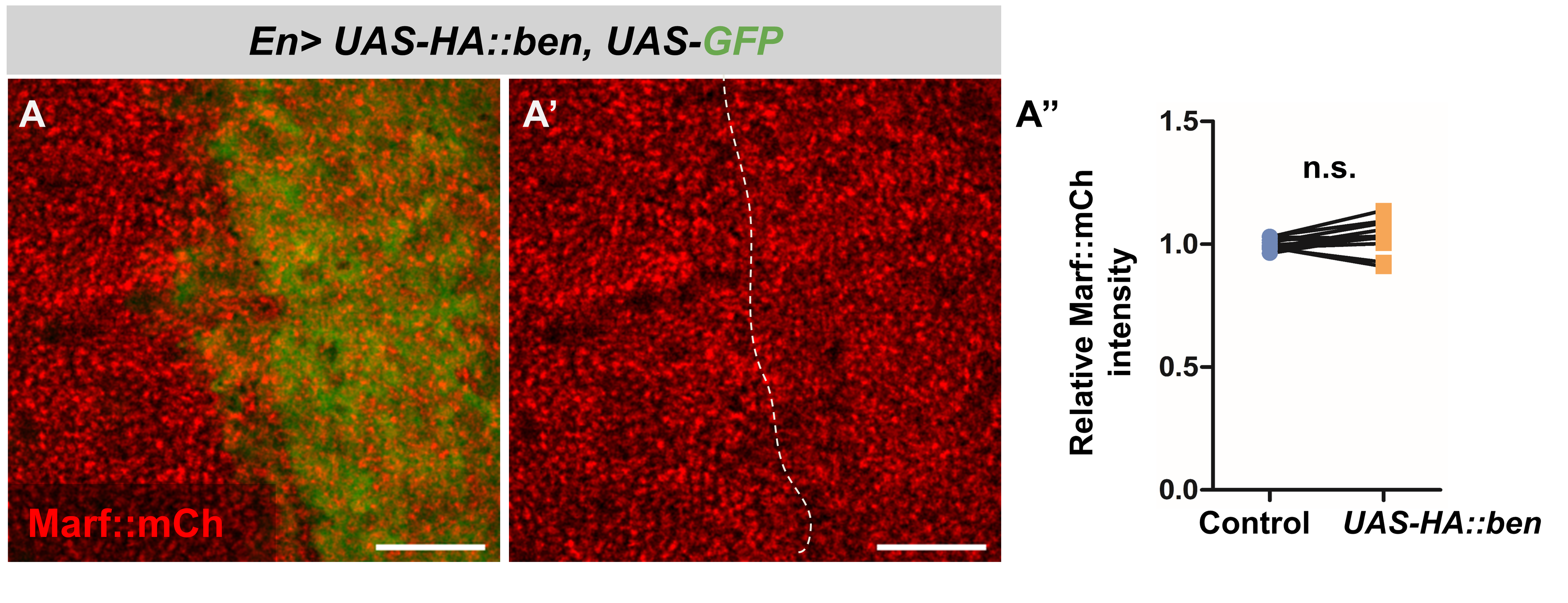

Supplement: S6 Fig — (A-A’) Wing discs immunostained for Marf::mCherry (red) on overexpression of HA::Ben using En>Gal4, wing discs marked with UAS-GFP (green). (A”) Quantification for relative fluorescence intensities of Marf::mCherry on overexpression of HA::ben (n = 12). Graphs represent average intensity values normalized to that of control cells. Two-tailed unpaired t-test between control and UAS-HA::Ben overexpressing cells. n.s—non significant. (TIF) [file pgen.1010493.s006.tif]

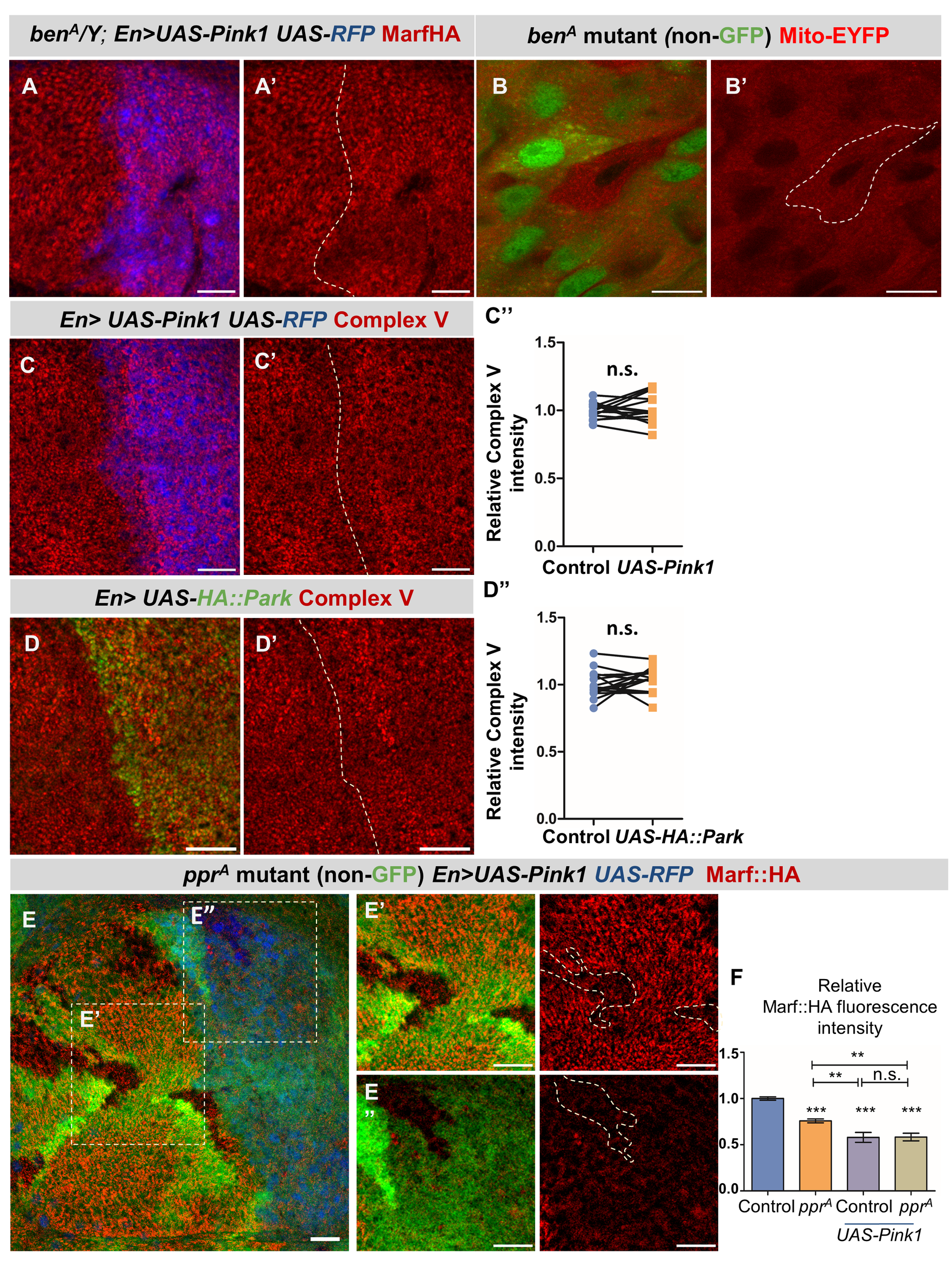

Supplement: S7 Fig — (A-A’) benA mutant on overexpression of Pink1 using En>Gal4, wing discs marked with UAS-RFP (blue) and immunostained for Marf::HA (red). (B-B’) benA mutant clone (non-green cell, B and dashed white line, B’), pupal gut 2h APF expressing Sq>mito-EYFP (red). (C-C’) Overexpression of Pink1 using En>Gal4, wing discs marked with UAS-RFP (blue) and immunostained for Complex V (red). (D-D’) Overexpression of Park using En>Gal4, wing discs immunostained for HA (green) and Complex V (red). Scale bar represents 20μm. (C” and D”) Quantification for relative fluorescence intensities of Complex V in UAS-Pink1 cells (C”, n = 15) and UAS-HA::Park cells (D”, n = 15). Graphs represent average intensity values normalized to that of control. Two-tailed unpaired t-test between control and cells overexpressing UAS-Pink1/UAS-HA::Park. (E-E”) Overexpression of Pink1 using En>Gal4, wing discs marked with UAS-RFP (blue, E) and immunostained for Marf::HA (red, E-E”) with lrpprc2A mutant clone (non-green cell, E, E’ and E” and dashed white line, E’ and E”). (F) Average Marf::HA intensity values in wildtype, lrpprc2A mutant clones, UAS-Pink1 and lrpprc2A mutant clones in UAS-Pink1 background, normalized to that of control cells (non RFP expressing GFP positive cells). A one-way ANOVA-Bonferroni’s multiple comparison test was used to calculate the significance between the samples in graph F. Error bars represent S.E.M. Significance represented by n.s.- non significant, p<0.05*, p<0.01**, p<0.0001***. (TIF) [file pgen.1010493.s007.tif]

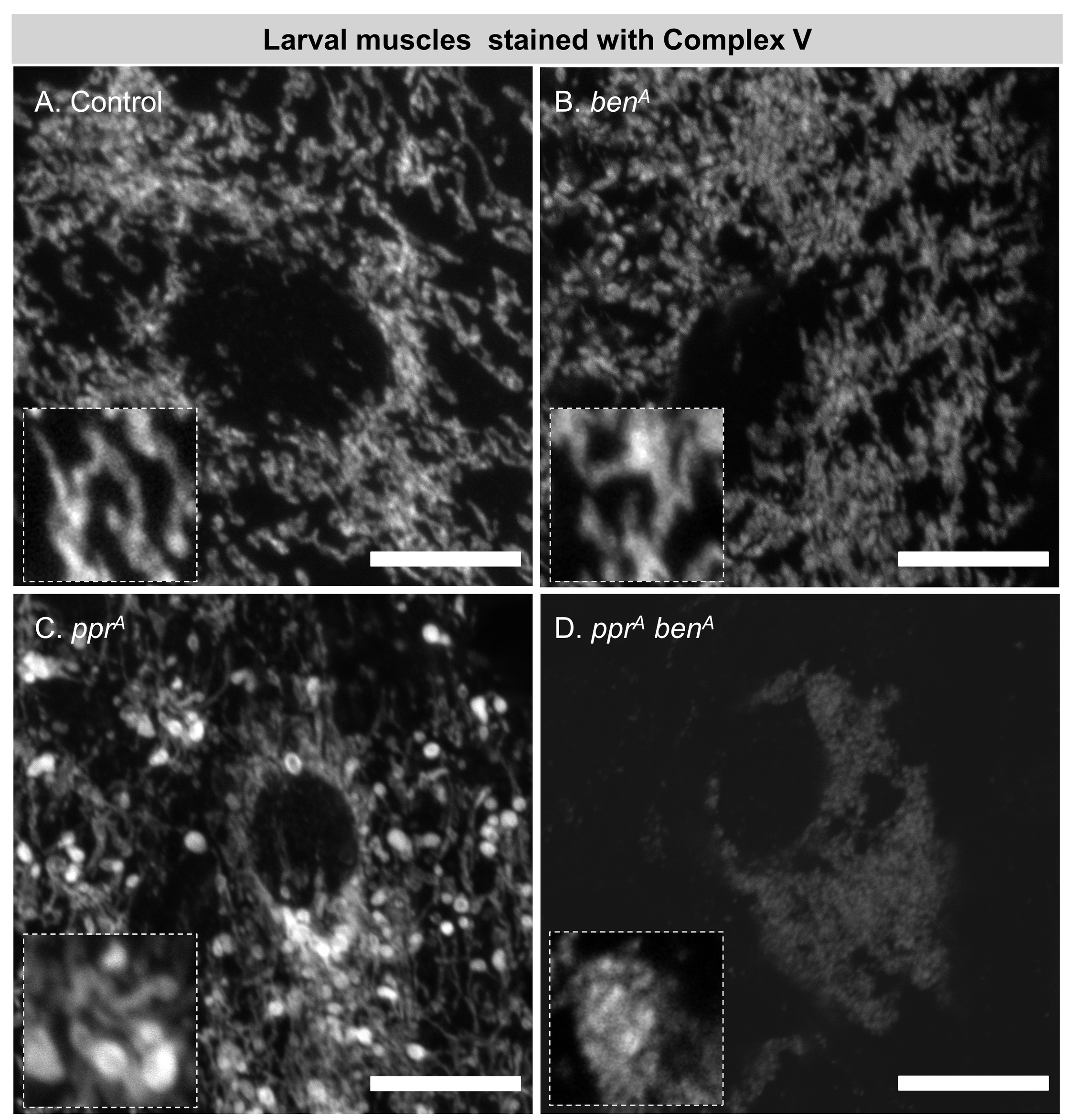

Supplement: S8 Fig — (A-D) Confocal sections of third instar larval muscles immunostained for endogenous Complex V (gray) in control(A), benA(B), lrpprc2A(C) and lrpprc2A benA(D) larvae. (TIF) [file pgen.1010493.s008.tif]

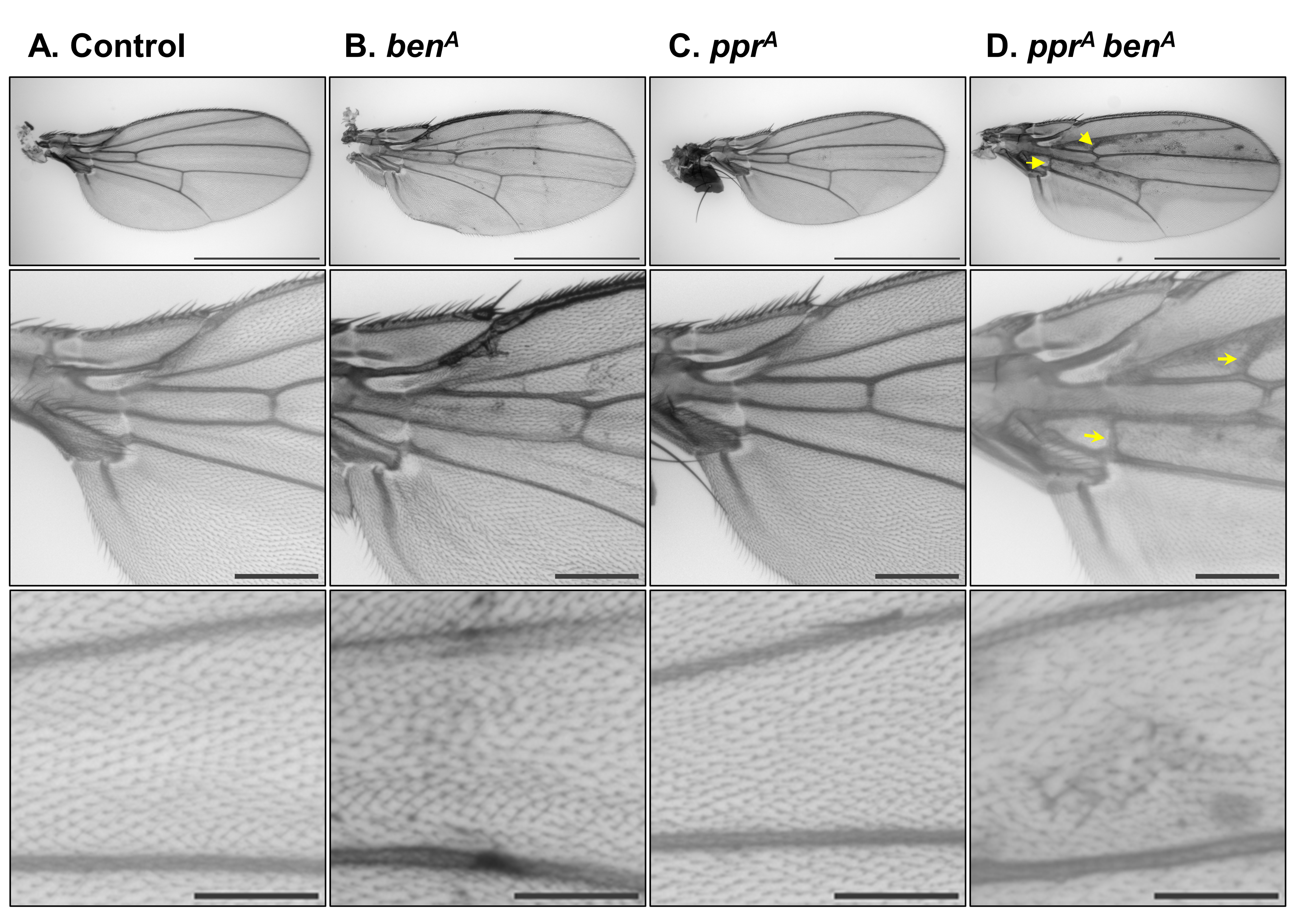

Supplement: S9 Fig — (A-D) Mutant wing clones from young flies of control(A-A”), benA(B-B”), lrpprc2A(C-C”), and lrpprc2A benA(D-D”) genotypes. Ectopic veins are marked by yellow arrowheads. Scale bar represents 1mm (A-D), 200μm (A’-D’) and 100μm (A”-D”) The images within the figure panels are created by the authors. (TIF) [file pgen.1010493.s009.tif]
